# Genome-wide association study reveals the genetic architecture for calcium accumulation in grains of hexaploid wheat (*Triticum aestivum L.*)

**DOI:** 10.1186/s12870-022-03602-z

**Published:** 2022-05-04

**Authors:** Xia Shi, Zhengfu Zhou, Wenxu Li, Maomao Qin, Pan Yang, Jinna Hou, Fangfang Huang, Zhensheng Lei, Zhengqing Wu, Jiansheng Wang

**Affiliations:** 1 Henan Institute of Crop Molecular Breeding, Henan Academy of Agricultural Sciences, Zhengzhou, 450002 China; 2 The Shennong Laboratory, Zhengzhou, 450002 Henan China; 3 College of Chemistry and Environment Engineering, Pingdingshan University, Pingdingshan, 467000 China; 4 College of Life Sciences, Henan Agricultural University, Zhengzhou, 450002 China

**Keywords:** Hexaploid wheat (*Triticum aestivum L.*), Ca accumulation, superior alleles, pyramid breeding, genome-wide association analysis

## Abstract

**Background:**

Hexaploid wheat (*Triticum aestivum L.*) is a leading cereal crop worldwide. Understanding the mechanism of calcium (Ca) accumulation in wheat is important to reduce the risk of human micronutrient deficiencies. However, the mechanisms of Ca accumulation in wheat grain are only partly understood.

**Results:**

Here, a genome-wide association study (GWAS) was performed to dissect the genetic basis of Ca accumulation in wheat grain using an association population consisting of 207 varieties, with phenotypic data from three locations. In total, 11 non-redundant genetic loci associated with Ca concentration were identified and they explained, on average, 9.61–26.93% of the phenotypic variation. Cultivars containing more superior alleles had increased grain Ca concentrations. Notably, four non-redundant loci were mutually verified by different statistical models in at least two environments, indicating their stability across different environments. Four putative candidate genes linked to Ca accumulation were revealed from the stable genetic loci. Among them, two genes, associated with the stable genetic loci on chromosomes 4A (AX-108912427) and 3B (AX-110922471), encode the subunits of *V-type Proton ATPase* (*TraesCS4A02G428900* and *TraesCS3B02G241000*), which annotated as the typical generators of a proton gradient that might be involved in Ca homeostasis in wheat grain.

**Conclusion:**

To identify genetic loci associated with Ca accumulation, we conducted GWAS on Ca concentrations and detected 11 genetic loci; whereas four genetic loci were stable across different environments. A genetic loci hot spot exists at the end of chromosome 4A and associated with the putative candidate gene *TraesCS4A02G428900*. The candidate gene *TraesCS4A02G428900* encodes *V-type proton ATPase subunit e* and highly expressed in wheat grains, and it possibly involved in Ca accumulation. This study increases our understanding of the genetic architecture of Ca accumulation in wheat grains, which is potentially helpful for wheat Ca biofortification pyramid breeding.

**Supplementary Information:**

The online version contains supplementary material available at 10.1186/s12870-022-03602-z.

## Background

Hexaploid wheat (*Triticum aestivum L.*) is one of the world’s three staple food crops, and its products are the main sources of human dietary nutrients [1]. In the past 50 years, with the continuous improvement of breeding and cultivation technology, the yield of major food crops has increased greatly, helping to solve the problem of food security [2]. The past breeding targets have focused too much on increasing yields and protein contents while ignored the development of nutritional quality, including that supplied by microelements [3]. However, with changes in consumers’ dietary structure and the continuous improvement in living standards, the demand for nutritional high-quality wheat is growing.

Ca is the fifth most required microelement, and its total content forms approximately 2% of the human’s body weight. It plays important roles in maintaining the human body’s normal physiological and biochemical functions, making it an important nutrient for human health [4]. Some basic regulatory functions in the human body involve Ca, including hormone secretion, blood clotting, enzyme reaction activation, vascular diastolic, muscle function, nerve impulse delivery, cell proliferation and intracellular metabolism [5]. The appropriate amount of Ca intake has protective effects on colorectal, ovarian, breast and other types of cancer, and it can effectively reduce the risk of cardiovascular disease [6, 7]. Moreover, Ca is also necessary for plant growth and development, due to its important roles in plant cell structures and physiological functions. It is involved in maintaining the stability of cell walls, cell membranes and membrane-binding proteins, and it participates in regulating intracellular homeostasis and plant development [8, 9]. As a second messenger in plant cell signaling, Ca has a sensitive response to various stimuli, such as environmental and endogenous hormone signals [9]. It participates in signal transduction processes that rely on concentration gradient changes to transmit signals [10]. Furthermore, depending on the Ca signal transduction genes, Ca also participates in biotic and abiotic stress responses, hormone regulation and other physiological and biochemical functions in plants [11, 12]. Therefore, both plants and humans require optimum intakes of Ca for their normal physiological and biochemical activities.

Ca elements can be present in the soil as free ions, or adsorbed onto mineral or organic surfaces as dissolved compounds or precipitates. Although Ca is enriched in soil, the phytoavailability of Ca elements is often restricted by the factors of ionic morphology, transport channel and transporters activity [13]. With the rapid development of molecular biology, there have been more and more reports on molecular mechanism for Ca accumulation. Previous studies revealed that proteins involved in Ca transport are mainly classified into three categories: *channel proteins*, *Ca*^*2+*^*-ATPases* and *Ca*^*2+*^*/H*^*+*^ *exchangers* [14]. Spatiotemporal regulation of Ca concentrations in the plant cells relies on the synergy of channels and active transporters on different organelles and cell membranes [15]. The cellular distribution and transport mechanisms of these transporters are distinct, and they are engaged in a complex and strict regulation network for Ca homeostasis in plants. Transmembrane diffusion of Ca (intracellular or plasma membranes) is mediated by Ca^2+^ channels, which include *cyclic nucleotide gated channels (CNGCs)*, *glutamate receptor like (GLR) proteins*, *two-pore channels (TPCs)* and *mechanosensitive Ca-permeable channels (MSCCs)* [16]. In addition, *Ca*^*2+*^*-ATPase* is a membrane-bound Ca^2+^ transporter which using the energy from the hydrolysis of ATP (adenosine triphosphate) to transport Ca^2+^ across membranes against a concentration gradient [17]. *Ca*^*2+*^*-ATPase* is mainly divided into two types: *P-type IIA* and *P-type IIB*, both of which play an important role in Ca^2+^ transport [18]. *P-type IIA* family proteins lack N-terminal autoregulation domains, while *P-type IIB* family proteins in plants contain N-terminal self-inhibiting domains, Ca^2+^/CAM binding sites and serine phosphorylation sites. *P-type IIA* and *P-type IIB* have been identified in cereal grains (such as finger millet and rice) and were strongly expressed at the later stage of grain development, facilitating Ca^2+^ to grain transport [19–21]. *Ca*^*2+*^*/H*^*+*^ *exchangers* is another type of Ca^2+^ secondary transporter that utilizes the energy of ion flow to actively transport Ca^2+^ reversely against its concentration gradient. It mainly distributes on the plasma membrane and vacuole membrane, and participates in Ca^2+^ transport in plants [20, 22, 23]. Although genes involved in the accumulation of Ca ions in grains have been identified in a variety of cereal crops, there are few reports in wheat, which restricts the molecular mechanism of Ca ions in wheat grains.

Quantitative trait loci (QTL) associated with Ca accumulation have been identified in different plant species and crops, such as wheat, rice, sorghum, barley, corn, pearl millet and beans [16, 24–26]. Goel et al. identified 31 QTLs in rice that regulate Ca accumulation and 28 QTLs in sorghum that affect Ca accumulation [23]. Five QTLs identified in Arabidopsis thaliana account for 36.4% of the Ca content variation [27]. However, QTL identification strategies using bi-parental populations have low resolutions and only relevant sites with residual variation at the parental level can be obtained. In contrast, a genome-wide association analysis (GWAS) relies on more representative and diverse natural population genome information to detect non-random associations between genotypes and phenotypes, greatly improving the resolution of the QTL mapping [28]. This method has been widely used in the genetic loci mapping of complex quantitative traits in multiple species. However, there are only a few studies in which GWAS has been used to identify loci associated with grain Ca accumulation in wheat. Alomari et al. used a natural population containing 353 wheat varieties to identify the major loci associated with the grain Ca accumulation efficiency on chromosomes 2A, 5B and 6A [29]. Bhatta et al. (2018) identified 14 genomic regions associated with Ca accumulation in grains on chromosomes 1B, 2B, 2D, 3A, 3B, 3D, 6A, 6B and 7A, which explained 2.7 to 21.5% of the phenotypic variation [30].

Previous studies have studied the accumulations of microelements in wheat grains using association and linkage analyses. However, there are limited reports on Ca accumulation in wheat grains; consequently, the genetic basis of Ca accumulation in wheat grains remains unclear. In this study, a natural population was employed to identify the genetic loci for wheat grain Ca accumulation using GWAS. This study aimed to 1) dissect the genetic architecture of wheat grain Ca accumulation; 2) identify potential candidate genes associated with Ca accumulation; 3) evaluate the genetic effects of stable non-redundant loci; and 4) explore molecular markers that can be used to guide Ca biofortification breeding.

## Results

### Genetic diversity analysis

**Fig. 1:**
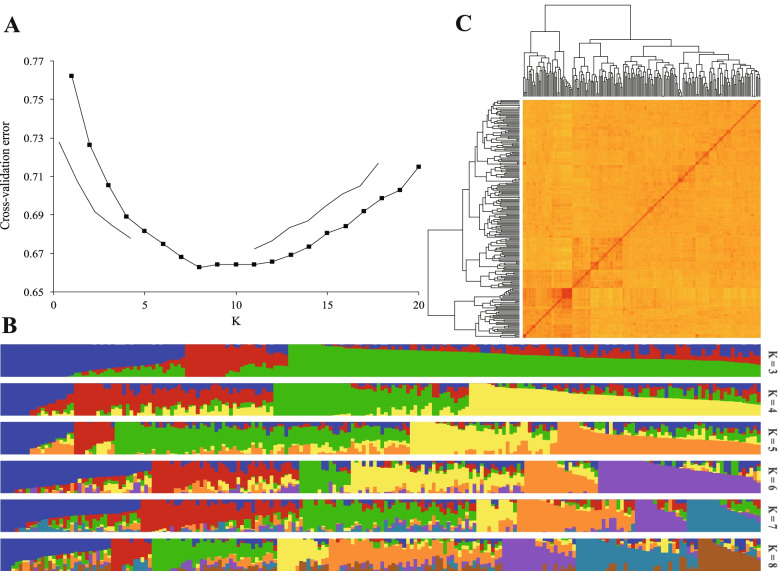
Population structure and kinship estimations in 207 wheat cultivars. **A** Plot of cross-validation errors. **B** Population structure of wheat cultivar panel from K = 3 to K = 8. **C** Kinship matrix, showing pairwise genetic relatedness among individuals

The wheat 660 K SNP assay was used for genotyping the natural population which including 207 lines. After filtering using the criteria of minor allele frequency > 0.05 and missing data <10%, 244,508 single-nucleotide polymorphism (SNP) markers were retained for further analyses (Fig. S1). The population structure results were integrated using Admixture software (www.genetics.ucla.edu/software/admixture). As shown in Fig. 1, at K = 8, the cross-validation error value was the lowest, indicating it was the optimal K value; this demonstrated that the natural population has a rich genetic background and could be divided into eight sub-populations. Thus, this population was suitable for further GWAS of wheat grain Ca accumulations. Finally, linkage disequilibrium (LD) was calculated for the whole genome and A, B, D sub-genome, respectively. The scatter plots of R^2^ against physical distance indicated a clear LD decay with increasing physical distance (Fig. S2). The LD decayed below R^2^ = 0.2, the highest LD was found in the B genome (25 Mb), followed by A (6 Mb) and D (5 Mb) genomes, while the whole genome LD decay distance was about 10 Mb.

### Phenotypic variations in Ca concentrations

**Fig. 2:**
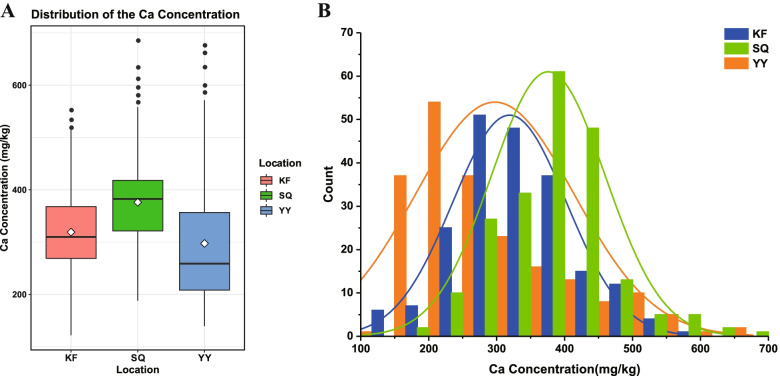
Distribution of the wheat grain calcium concentrations measured in the natural population. **A** Boxplot showing the calcium concentrations among different locations. Data from three locations, Yuanyang (YY), Kaifeng (KF) and Shangqiu (SQ), are shown. B Distribution of the calcium concentrations in the association populations from YY, KF and SQ.

The Ca levels were investigated for the whole natural wheat population across field studies in Yuanyang (YY), Shangqiu (SQ) and Kaifeng (KF) in 2017 (Table S1). In each environment, the Ca concentration showed a broad range of variation (Fig. 2). The highest mean Ca concentration (376.02 mg/kg) was recorded in SQ, where values ranged from 187.87 to 685.17 mg/kg. The second highest mean Ca concentration was 319.05 mg/kg recorded in KF, where values ranged from 121.71 to 552.19 mg/kg. A lower mean Ca concentration value of 297.37 mg/kg was recorded in YY, where the values ranged from 139.18 to 676.04 mg/kg. The resulting of the best linear unbiased predictors (BLUPs) for grain Ca concentrations across all environments ranged from 320.96 to 348.08 mg/kg, with a mean of 330.81 mg/kg (Table 1). Additionally, the frequencies of the Ca concentrations in individual and BLUP environments exhibited an approximately normal distribution (Fig. S3). Thus, this is an ideal population for GWAS of wheat grain Ca concentrations.

**Table 1:** Descriptive statistics of the Ca concentrations in the association population

Location	Trait	Mean ± SD^a^ (mg/kg)	Range (mg/kg)	Kurt^**b**^	Skew^**c**^
Yuanyang (YY)	Ca concentration	297.37 ± 116.77	139.18–676.04	0.5	1.1
Kaifeng (KF)	Ca concentration	319.05 ± 83.60	121.71–552.19	0.06	0.26
Shangqiu (SQ)	Ca concentration	376.02 ± 83.87	187.87–685.17	0.92	0.39
BLUP	Ca concentration	330.81 ± 5.37	320.96–348.08	0.7	1.01

^a^ *SD* standard deviation

^b^ Kurt, kurtosis, which is a measure of the ‘tailedness’ of the probability distribution of a real-valued random variable

^c^ Skew, skewness, which is a measure of the asymmetry of the probability distribution of a real-valued random variable about its mean

### GWAS of wheat grain Ca concentrations

In order to improve the confidence of the marker-trait association (MTA) analysis, three models, general linear models (GLM), mixed linear models (MLM) and fixed and random model Circulating Probability Unification (FarmCPU), were used to perform the GWAS for the Ca concentration in wheat grains at the three locations and the BLUP. Totally, 283 significant SNPs at the suggested *p* < 1.0 × 10^−4^ for wheat grain Ca concentrations were identified in the surveyed environments (including the BLUP) (Fig. S4). Among them, seventy-five significant SNPs for wheat grain Ca concentrations were identified by all three model (MLM, GLM and FarmCPU) (Fig. S4), which categorized into 11 loci and were considered to be more reliable (Table 2). These loci were mainly distributed on chromosomes 2A, 3A (2 loci), 3B (2 loci), 3D, 4A, 4B, 5B (2 loci) and 6A, respectively, and the PVE ranged from 9.66 to 26.93%. Among them, 4 loci involving 4 peak SNPs were detected in at least two environments, and they were stable across different environments (Table 2). Additionally, the SNP AX-108912427 was identified simultaneously in all environments by MLM statistical model and was detected in three environments by GLM and FarmCPU statistical models, respectively (Table S2–4). It was located on chromosome 4A and exhibited the highest PVE in each environment, ranging from 12.89 to 26.93% (Table 2). Furthermore, a QTL hot spot exists at the end of chromosome 4A. This locus could be a key factor in regulating wheat grain Ca accumulation.

**Table 2:** List of significant loci and their detailed information for Ca accumulation identified by GLM, MLM and FarmCPU models

ID^**a**^	Chr.^**b**^	Interval (bp)	No. of SNPs^**c**^	Location	Peak SNP^**d**^	Position (bp)	***P*** value^*^	R^**2**^(%)^**e**^
1	2A	49,069,207–49,219,472	20	KF	AX-110634514	49,219,472	2.76E-05	16.10
				YY	AX-110634514	49,219,472	7.01E-05	13.89
2	3A	38,138,188–38,141,372	2	BLUP	AX-111799835	38,138,188	5.27E-05	17.09
3	3A	662,398,903	1	SQ	AX-109541359	662,398,903	6.77E-05	9.66
				YY	AX-109541359	662,398,903	1.77E-05	14.91
4	3B	2,151,413–20,504,151	7	YY	AX-110013515	13,967,085	2.33E-05	15.03
				BLUP	AX-110013515	13,967,085	3.33E-05	18.03
5	3B	376,625,452–404,801,032	2	YY	AX-110922471	376,625,452	1.71E-09	24.81
				BLUP	AX-110922471	376,625,452	5.56E-05	17.04
6	3D	40,526,440	1	SQ	AX-94729264	40,526,440	1.05E-05	11.55
				YY	AX-94729264	40,526,440	2.65E-13	26.80
				BLUP	AX-94729264	40,526,440	1.68E-05	18.15
7	4A	676,781,724–699,571,654	34	KF	AX-108912427	699,571,654	2.35E-05	16.55
				SQ	AX-108912427	699,571,654	2.87E-06	12.89
				YY	AX-108912427	699,571,654	2.24E-13	26.93
				BLUP	AX-108912427	699,571,654	3.83E-06	19.55
8	4B	10,703,582–10,703,651	2	SQ	AX-110637930	10,703,582	3.79E-05	10.24
9	5B	431,075,720	1	YY	AX-108753683	431,075,720	8.71E-05	13.77
10	5B	606,299,294–606,814,678	4	SQ	AX-111141605	606,299,294	5.00E-05	9.96
11	6A	520,873,173	1	YY	AX-111183340	520,873,173	2.88E-05	14.83

^*^The *p*-values were calculated by the MLM model

^a^ The ID of the loci identified in the GWAS

^b^ Chromosome

^c^ The number of significant SNPs

^d^ Most significant SNP

^e^ Percentage of phenotypic variance explained by the MTA from the results of MLM model

**Fig. 3:**
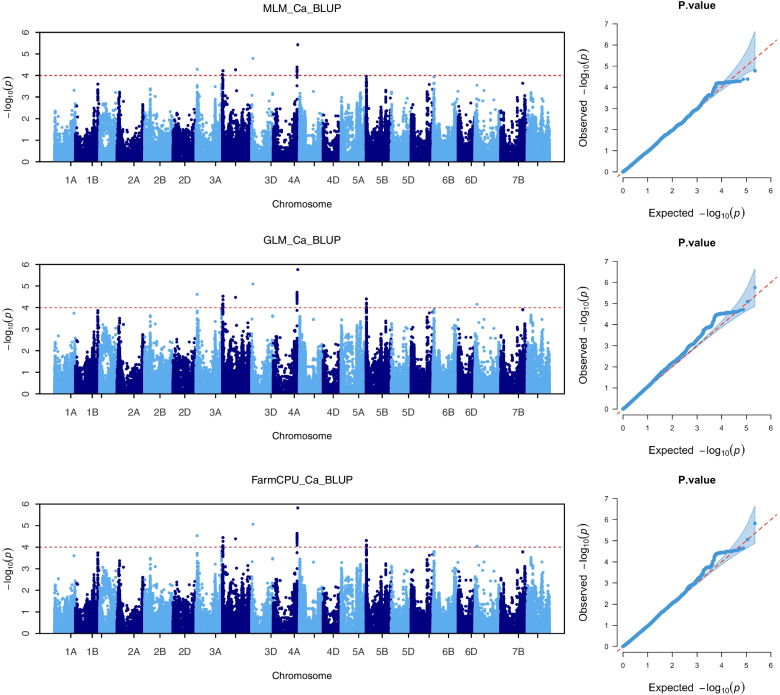
Manhattan and quantile-quantile plots for wheat grains Ca concentrations using GLM, MLM and FarmCPU models in BLUP. The dashed horizontal line represents the significant threshold of −log_10_(P) = 4.0. The SNPs above the red dotted line are significantly associated with Ca variation

Manhattan plot and quantile–quantile (QQ) plot of GLM, MLM and FarmCPU statistical models of wheat grain Ca accumulation in BLUP environment are shown in Fig. 3. The remaining environments of the GLM, MLM and FarmCPU models manhattan plot and QQ plot are presented in Fig. S5-S7, respectively. Significant SNPs related information of GLM, MLM and FarmCPU models were listed in Table S2-S4, respectively.

### Prediction of the candidate genes for stable genetic loci controlling Ca accumulation

Four peak SNPs, together with corresponding loci associated with Ca accumulation were identified by the GWAS with different statistical models in multiple environments in this study. Before the candidate gene prediction, we compared the QTLs detected by the present study with previous research on Ca accumulation in wheat and found these loci co-localize (Table 3). The recent annotated wheat genome reference sequence (IWGSC RefSeq v2.0) and the Wheat Expression Browser (http://www.wheat-expression.com) public database were employed to identify the candidate genes possibly associated with the four stable genetic loci for Ca accumulation. We screened 34, 28, 50 and 22 genes within 10 Mb of the sequence flanking loci (5 Mb upstream and downstream) of AX-110013515, AX-110922471, AX-94729264 and AX-108912427 locus, respectively (Table S5). Interestingly, most genes were highly expressed in roots, leaves/shoots or spike; while only 18 genes were highly expressed in grains and had a higher expression level in grains than other tissues (Fig. S8). Notably, the most significant SNP AX-108912427 is physically closest to the candidate gene *TraesCS4A02G428900*, and it located in the 3’UTR region of *TraesCS4A02G428900* on chromosome 4A. This gene encodes a *V-type proton ATPase subunit e*, which may relevant to Ca homeostasis and transporters. Nearby the second most significant SNP AX-110922471 on chromosome 3B, only one gene was highly expressed in grain and its expression level was significantly higher than in other tissues, and this gene encodes *V-type proton ATPase subunit d* as an important component of V-type proton ATPase. Additionally, two SNPs, AX-110013515 and AX-94729264, located on chromosomes 3B and 3D, respectively, were linked with the predicted genes *TraesCS3B02G019900* and *TraesCS3D02G079600*, respectively. The first gene encodes a *histidine-rich calcium-binding protein*, and the second gene encodes a *ubiquitin family protein*, which may be relevant to Ca regulation in wheat grains. Integrating the analysis of gene annotation information, genes physical position and the gene expression profiles, the above four promising genes were considered as the candidate genes relevant to Ca accumulation in wheat grains. Detailed information on the most likely candidate genes is list in Table 3.

**Table 3:** Information for stable genetic loci associated with Ca accumulation identnfied via GWAS

SNP_id^**a**^	Chromosome	Position (Mb)^**b**^	Region	Near locus previous reported in the same chromosome^**c**^	Candidate genes	Annotation
			(Mb)			
AX-110013515	3B	13.97	2.15–20.50	*gwm389-wPt-8093(C)*	*TraesCS3B02G019900*	*Histidine-rich calcium-binding protein*
AX-110922471	3B	376.63	294.5–404.8	*QGCaUE-3B*	*TraesCS3B02G241000*	*V-type proton ATPase subunit d*
AX-94729264	3D	40.53	40.53	*S3D_45073985*	*TraesCS3D02G079600*	*Ubiquitin family protein*
AX-108912427	4A	699.57	676.78–699.57	*gwm165a-wmc420*	*TraesCS4A02G428900*	*V-type proton ATPase subunit e*

^a^ The ID of the loci linked to the significant SNP identified in the GWAS

^b^ The significant SNPs’ physical positions in the ‘Chinese Spring’ reference genome (IWGSC RefSeq v2.0)

^c^ Co-localized QTLs at this locus compared with in the literature

### Relationship between Ca accumulation and the number of superior alleles

**Fig. 4:**
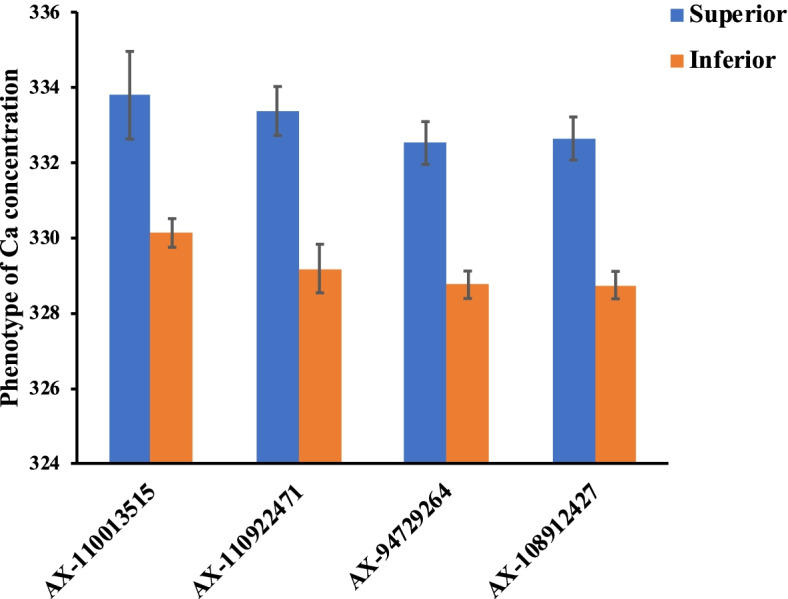
The phenotype values of accessions with superior alleles (blue bar) and inferior alleles (orange bar) of repetitive significant SNPs for wheat grain Ca concentration

All these 4 peak SNPs identified by GWAS with different statistical models in at least two environments were considered as the stable loci for controlling Ca accumulations in wheat grains. The repeatable SNP polymorphic effects were investigated using the analysis of variance (ANOVA) method based on the phenotypic values of the natural population. For each of the four SNPs, cultivars with the superior alleles showed higher Ca concentration in grains than cultivars with the inferior alleles (Fig. 4). Statistical analyses showed that the grain Ca concentration differences, accessions with superior alleles compared with the accessions with inferior alleles, have reached significant or highly significant levels in all or some environments (Table 4).

**Table 4:** ANOVA for individuals harboring the superior and inferior alleles for the stable significant SNPs in multi-environments

SNP_id	Chr.	Allele type		Phenotype value(BLUP)		Allele number		Allele percentage(%)		*p*-Value			
		Superior	Inferior	Superior	Inferior	Superior	Inferior	Superior	Inferior	Ca_YY	Ca_SQ	Ca_KF	Ca_BLUP
AX-110013515	3B	AG	AA	333.79	330.13	33	169	16.34	83.66	1.31E-06	5.35E-01	1.82E-01	2.97E-04
AX-110922471	3B	GG	AA	333.37	329.18	86	54	61.43	38.57	5.04E-06	6.91E-01	8.85E-04	2.52E-05
AX-94729264	3D	CC	CT	332.52	328.75	113	94	54.59	45.41	2.35E-07	1.22E-06	6.46E-04	2.34E-07
AX-108912427	4A	GG	AG	332.64	328.74	111	94	54.15	45.85	7.41E-21	3.73E-06	7.04E-04	9.08E-08

**Fig. 5:**
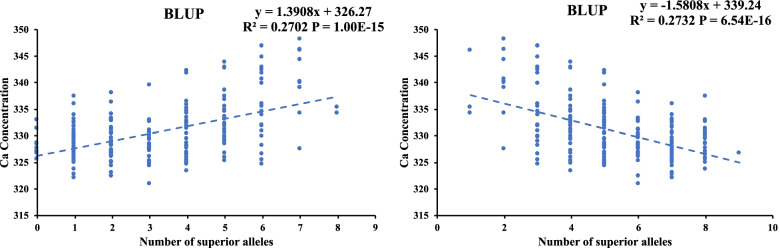
Linear regression between the number of (**A**) superior alleles and (**B**) inferior alleles

To further understand the pyramiding effects of all the 11 detected loci on the grain Ca concentration, we investigated the numbers of superior and inferior alleles in each cultivar. The number of superior alleles ranged from 0 to 8, compared with 1 to 9 inferior alleles (Table S6). The Ca concentration-related BLUP value of the natural population was used to examine the relationship between Ca accumulation and the numbers of superior and inferior alleles by linear regression. A linear relationship between grain Ca concentration and the number of superior alleles per genotype was observed. The regression coefficients between the Ca concentration and numbers of superior alleles and inferior alleles were 0.2702 and 0.2732, respectively, implying that superior alleles contributed to increasing grain Ca concentrations (Fig. 5). Thus, pyramiding more superior alleles should enhance the wheat grain Ca concentration, and this strategy can be used in crop genetic biofortification breeding programs.

## Discussion

Genetic biofortification is an effective method for enhancing crop microelement contents. A large number of genetic loci controlling microelement accumulation in wheat, rice and other crops have been identified in previous research [31–34]. However, there are relatively few studies on the genetic mechanisms of Ca ion accumulation in wheat grain; consequently, limited information is available on wheat grain genetic control and molecular physiological mechanisms. GWAS is a powerful tool for dissecting the genetics of complex traits and identifying the chromosomal regions harboring genes suitable for use in breeding programs. In this study, a GWAS was used to dissect the genetic basis of Ca accumulation in wheat grain using a natural population.

### High-density molecular markers and genetic diversity

When using a GWAS, the probability of detecting the causal variant and associated loci for a target trait depends on the marker density, population size and statistical methods [35]. Owing to the rapid development and application of molecular marker assays, Wheat 35 K, 90 K, 660 K and 820 K SNP genotyping arrays have been designed and utilized for GWAS and linkage analyses in common wheat [36–38]. Comparative analyses revealed that the Wheat 660 K SNP array is reliable and cost-effective, making it the ideal choice for genotyping a population of individuals [39]. In the present study, a credible number of markers (244,508 SNPs) was identified using the Wheat 660 K SNP array, and the population met the requirements of a GWAS for Ca accumulation in wheat grains. Population size is another factor limiting the detection efficiency of a GWAS. The effect of increasing the population size on loci detection efficiency is greater than that of the marker density [40], and increasing the population size may lead to the identification of more smaller-effect QTLs [41]. Previously, population sizes ranging from 100 to 500 have been used for wheat association analyses [42–44]. In this study, although the natural population of 207 diverse accessions was not sufficiently large, the dramatic phenotypic variations in grain Ca concentration was very large, ranging from 121.71 mg/kg to 685.17 mg/kg, and it showed a normal distribution, which is conducive to GWAS. Dramatic phenotype variation may be associated with high genetic diversity [45].

### Ca grain concentration is controlled by multiple loci

The manifestations of complex quantitative traits (such as microelement accumulation in wheat grain) are often controlled by multiple genetic loci [30, 46, 47]. Several genetic loci affecting Ca accumulation in wheat grain were identified previously by GWAS and linkage analyses [24, 29, 30], which allowed for a comparison among loci identified in known QTLs and those identified in the present study. Here, 18 non-redundant loci for Ca accumulation were distributed on chromosomes 1D, 2A, 2B, 3A, 3B, 3D, 4A, 4B, 5A, 5B, 6A, 7A and 7B, which suggested the involvement of loci on these chromosomes in the natural population regulating Ca concentration variation. QTLs for Ca accumulation are scattered on chromosomes 4A, 4B, 5B, 6A and 7B [24] in a *durum wheat* × *wild emmer* RIL population, which overlapped with loci identified in the current study, indicating that the linkage mapping results were complementary to those of the GWAS for wheat grain Ca accumulation. Bhatta et al. (2018) identified 15 significant marker-trait associations for wheat grain Ca accumulation distributed in 14 different genomic regions on chromosomes 1B, 2B, 2D, 3A, 3B, 3D, 6A, 6B and 7A that explained 2.7 to 21.5% phenotypic variation using the Hard Winter Wheat Association Mapping Panel (including 299 varieties) and the GWAS method [30]. We only identified eight loci on chromosomes 2B, 3A (2 loci), 3B (2 loci), 3D, 6A and 7A, and not all of the loci detected previously were identified in this study. This may be because of (a) the different origins of the populations, or (b) the use of different genotypic identification platforms. This can make it difficult to align the complete genomes of the population’s individuals. It is worth noting that, like other complex quantitative traits, the accumulation of Ca in wheat grain is controlled by multiple genetic sites and is susceptible to environmental influences [48]. Therefore, the ideal target genetic loci should be stably identified under multi-environmental conditions. In this study, we found that all four loci were detected in at least two environments with relatively higher PVE values (9.66–26.93%), which suggests that these were stable QTLs significantly associated with wheat grain Ca accumulation that were critical for target trait phenotypic variation. On chromosome 3B, the SNPs AX-110013515 and AX-110922471 were mapped to genomic regions near *gwm389-wPt-8093(C)* and *QGCaUE-3B*, respectively. The former locus has been identified in a natural wheat population [49], and the latter locus has been detected in a double-haploid wheat population [47]. The peak SNP AX-94729264 was simultaneously identified in SQ, YY, and BLUP, which indicated it co-localizes with *S3D_45073985* [30]. These findings validate the results of the GWAS and increase the confidence in some loci identified in the present study. The hotspot at the end of chromosome 4A linked with SNP AX-108912427 (identified in all the environments with the highest PVE values, ranging from 12.89 to 26.93%) was simultaneously mapped in the vicinities of three QTLs, *gwm165a-wmc420*, *wmc106-gwm165a* and *gwm610* [24, 49], which implies that this hotspot is a key factor that harbors a major gene for regulating Ca accumulation in wheat grain.

### Putative candidate genes for Ca accumulation

In this study, four peak SNPs, together with corresponding loci associated Ca accumulation, were identified by GWAS with different statistical models in multiple environments (Table S2–4). Combined with the physical position, functional annotation information and gene expression pattern (Fig. S8), four genes were identified as the most credible candidate genes for Ca accumulation (Table 3). Two SNP markers, AX-108912427 on chromosome 4A and AX-110922471 on chromosome 3B, were associated with genes encoding *V-type proton ATPase subunit e* (*TraesCS4A02G428900*) and *V-type proton ATPase subunit d* (*TraesCS3B02G241000*), respectively. Both subunits are important components of the V-type proton ATPase that is the typical generator of a proton gradient involved in Ca ion sequestration in plant cells, and it may influence Ca ion homeostasis in wheat grains [50, 51]. The hot spot locus linked with 39 significant SNPs at the end of the 4A chromosome. The peak SNP, AX-108912427, has the highest average PVE at 21% for Ca concentration and was identified in all the environments, which implies that V-type proton ATPase is a key factor affecting Ca accumulation in wheat grain. Another marker on chromosome 3B, AX-110013515, corresponds to a *histidine-rich calcium-binding protein* (*TraesCS3B02G019900*). This protein initially identified in many mammalian species, including rabbits, humans, mice, rats and monkeys [52–55], and served as the regulators of Ca ion uptake, storage and release [56]. However, recent study about the homolog in *L. chinensis* found this gene might had distinct Ca ion binding sites and could interact with a histone deacetylation protein to mediate the transient rises for Ca ion concentration in plant nuclei [57]. Additionally, overexpressing the nucleus-localized Ca ion binding histidine-rich calcium-binding protein could alter Ca ion homeostasis in Arabidopsis nucleus [58]. The above study implied that *TraesCS3B02G019900* gene may closely associated with Ca accumulation in wheat grain. The remaining SNP AX-94729264 on chromosome 3D, were associated with candidate gene *TraesCS3D02G079600*. This gene encodes a *ubiquitin family protein* involved in the process of ubiquitin conjugation. Mutating ubiquitin proteins may alter cell coupling and the resulting Ca elevation [59], which implies that ubiquitin family proteins may participate in altering Ca homeostasis in wheat. Notably, all the above-mentioned candidate genes were highly expressed in grains and demonstrated higher expression level in grains than other tissues (Fig. S8). Although our findings provide clues for the molecular mechanisms underlying the complex nature of Ca accumulation in wheat grain, this topic requires further investigation.

### Application of MTAs for pyramid breeding

Organismal Ca requirements must be met through dietary uptake. The Ca intake in the adult population of Asia has been reported to be less than 500 mg/day, and in Africa and South America the Ca intake of the adult population is between 400 and 700 mg/day [16]. These values are far below the recommended standards of the Food and Agriculture Organization, which are 1300 mg/day for children over 9 years of age and 800–1300 mg/day for adults. Biofortification is an effective strategy to increase the microelement content of wheat and improve the human intake of Ca. However, early breeding programs mainly focused on yield and ignored the microelement levels. Because of the so-called “dilution effect” in which high yields are negatively correlated with micro-element levels [3], it is difficult to select wheat varieties with high Ca contents at high yield levels using traditional breeding methods. Identifying superior allele loci and developing corresponding molecular markers have been beneficial to pyramid breeding, and this strategy could significantly enhance the microelement, including Ca, levels in wheat grain [60–62]. In this study, four stable loci were identified as harboring superior alleles and exhibited significantly higher Ca accumulations in two or more environments. By comparing the abilities of lines with both superior and inferior alleles for Ca accumulation in wheat grain at the four stable loci, we found that phenotypic differences reached significant levels between individuals with either superior or inferior alleles (Fig. 4, Table 4). Additionally, a linear regression showed that with an increase in the number of superior alleles, the Ca concentrations of accessions gradually increased, revealing a significant additive effect, which provides guidance for pyramid breeding (Fig. 5). Markers identified by the GWAS that were significantly linked to these loci and associated with wheat grain Ca concentration may be converted into competitive allele-specific PCR markers for molecular marker-assisted selection-based breeding programs [63]. Using marker-assisted selection, the superior alleles for Ca accumulation could be integrated for multi-loci pyramid breeding, which will provide guidance for biofortification breeding. In the future, our studies will focus on validating the effects of these loci and developing molecular markers for wheat Ca biofortification pyramid breeding.

## Conclusion

In the present study, 11 non-redundant loci associated with Ca concentration were identified in the surveyed environments by three models (GLM, MLM and FarmCPU) and 4 non-redundant loci were stable across different environments. Among them, a hot spot exists at the end of chromosome 4A and exhibited the highest PVE in each environment, ranging from 12.89 to 26.93%. It is implied that this locus may be embraced a key factor in regulating wheat grain Ca accumulation. Haplotype analysis results showed that cultivars containing more superior alleles had increased grain Ca concentrations, which can be used for marker-assisted selection for varieties with high Ca concentrations in wheat grain at the early developmental stages without needing to phenotype mature plants. This study not only increases our understanding of the genetic architecture of grain Ca accumulation in wheat, but also provide a guidance for wheat Ca biofortification pyramid breeding.

## Materials and methods

### Plant material

A natural wheat population, consisting of 207 representative varieties that collected from the Henan Province Crop Germplasm Bank and The International Maize and Wheat Improvement Center (CIMMYT), was used in the GWAS for Ca accumulation in wheat grain and the name of individual varieties were listed in Table S1. The natural population was grown under three environments at Yuanyang (YY; E113°97′, N35°5′), Kaifeng (KF; E114°30′, N34°80′) and Shangqiu (SQ; E115°65′, N34°45′) in northern China during the 2016–2017 cropping season (October 2016 to June 2017). The average annual temperatures were 14.3 °C, 15.0 °C and 14.2 °C, and the average annual rainfalls were 556, 656 and 623 mm, in YY, KF and SQ, respectively. The natural population was separately sown in different plots of 1.0 m × 1.5 m containing four rows, with 10-cm spaces between individuals and 23 cm spaces between rows. The natural population at each location was subjected to standard agronomic practices.

### Phenotypic analysis of wheat grains with different Ca concentrations

The whole natural population, including 207 cultivars, was harvested after reaching physiological maturity (8–10% moisture content) at the three different locations. Grain samples (approximately 50 g/cultivar) were threshed by hand and carefully cleaned, and broken grains and sundries were removed. The samples were stored in paper sacks for the micronutrient analysis. The samples were milled using a Retch mill (MM301, Germany) and were dried overnight at 40 °C. Then, 0.5 g dried powder samples from each cultivar were digested with 5 mL nitric acid (HNO_3_, 69%, analytical reagent grade, Merck, Darmstadt, Germany) using a microwave reactor (UltraCLAVE, Milestone, Germany). After cooling, digested samples were adjusted to 25 mL with de-ionized distilled water (Milli-Q Reference System, Merck, Germany). The Ca concentration was measured using inductively coupled plasma–mass spectrometry (Agilent 7800, Agilent Technologies Inc., USA). Each sample was tested three times to generate technical replicates, and the average values were used for further analyses.

### Genotyping and quality control

For each accession, total genomic DNA was extracted from young leaf tissue using the CTAB (cetyltrimethylammonium bromide) procedure [64]. The 207 accessions were genotyped using the Wheat Breeders 660 K Axiom® array with the Axiom 2.0 Assay Manual Workflow protocol [37]. The accuracy of SNPs was determined using Plink version 1.9 software (http://www.cog-genomics.org/plink2/) with the criteria of minor allele frequency > 0.05 and missing genotype data <10%. In total, 244,508 SNPs were considered as creditable markers for further analyses (Fig. S1).

### GWAS mapping

Before performing the GWAS, the population structure (Q) and kinship (K) matrices were analyzed using ADMIXTURE version 1.3.0 (www.genetics.ucla.edu/software/admixture) and the GAPIT package [65] in R software (R Core Team, 2020), respectively. An admixture model with 10 replicates for each genetic group (K = 1–10) was implemented. A burn-in of 1000 iterations followed by 1000 Markov chain Monte Carlo replicates was used to estimate the number of subpopulations. After the operation, the different cross-validation error values were produced, and the optimal K was considered as having the minimal cross-validation error value. The natural population K matrix was calculated using the VanRaden method to determine relative kinship among the sampled individuals [66]. The values for BLUPs of Ca concentration, according to variety and location (YY, KF and SQ), were calculated using the mixed linear model in R package “lem4”, and they were computed using the following formula: Y = (1|Line) + (1|Loc) + (1|Rep%in%Line: Loc) + (1|Line: Loc).

The GWAS analysis for wheat grain Ca accumulations was performed using the R package GAPIT [56]. To select the optimal statistical model, three models, GLM (only accounts for population structure), MLM (accounts for population structure and relative kinship) and FarmCPU (accounts for fixed and random effects and improves calculation speed and accuracy), were used to analyze the association between phenotypic and genotypic datasets. The suggested *p*-value for significance was 1.0 × 10^−4^ to control the genetic false positive error rate for this population [67].

### Putative candidate gene predictions

Based on the reported common wheat variety Chinese Spring’s reference genome sequence “IWGSC RefSeq v2.0”, a high confidence gene list was downloaded from IWGSC (http://www.wheatgenome.org/) and used to identify putative candidate genes in each locus. Candidate genes were annotated using Ensemble Plants (http://plants.ensembl.org/Triticum_aestivum/Info/Index). The physical position of each SNP from the 660 K arrays was obtained from the IWGSC website (http://www.wheatgenome.org/Tools-and-Resources/IWGSC-RefSeq-v2.0). For each locus without appropriate candidates, the gene nearest to the peak SNP was assigned.

## Supplementary Information

**Additional file 1: Figure S1.** Single nucleotide polymorphism (SNP) density (number of SNPs within 1 Mb window size) of 207 bread wheat lines analyzed with the wheat 660 K SNP assay. **Figure S2.** LD decay distance estimated for 207 wheat accessions. **Figure S3.** Histogram of the calcium concentrations in wheat grains. Data from BLUP environment are shown. **Figure S4.** Venn diagram of significant SNPs associated with wheat grains Ca accumulation were identified by three GLM, MLM and FarmCPU models. **Figure S5.** Manhattan and quantile-quantile plots for Ca concentrations using the GLM for wheat grains across different environments (including BLUP). The dashed horizontal line represents the significant threshold of −log_10_(P) = 4.0. The SNPs above the red dotted line are significantly associated with calcium variation. **Figure S6.** Manhattan and quantile-quantile plots for Ca concentrations using the MLM for wheat grains across different environments (including BLUP). The dashed horizontal line represents the significant threshold of −log_10_(P) = 4.0. The SNPs above the red dotted line are significantly associated with calcium variation. **Figure S7.** Manhattan and quantile-quantile plots for Ca concentrations using the FarmCPU for wheat grains across different environments (including BLUP). The dashed horizontal line represents the significant threshold of −log_10_(P) = 4.0. The SNPs above the red dotted line are significantly associated with calcium variation. **Figure S8.** Expression level of candidate genes in different wheat tissues. The heat map was plotted using the transcripts per kilobase million (TPM) values after log_2_ conversion, which were obtained from the public database of Wheat Expression Browser (http://www.wheat-expression.com). (A) the heat map of high-confidence candidate genes within 10 Mb physical intervals from the SNPs AX-110013515, (B) AX-110922471, (C) AX-94729264 and (D) AX-108912427, respectively. **Table S1.** Average phenotypic values of Ca accumulation in 207 wheat accessions across from each environments and BLUP. **Table S2.** Marker-trait associations for Ca accumulation in the associated population analyzed by GLM model. **Table S3.** Marker-trait associations for Ca accumulation in the associated population analyzed by MLM model. **Table S4.** Marker-trait associations for Ca accumulation in the associated population analyzed by the FarmCPU model. **Table S5.** The expression values of high-confidence candidate genes within 10 M physical intervals of the 4 stable loci identified by GWAS in different wheat tissues. **Table S6.** Number of superior and inferior alleles across 11 significantly associated SNPs identified by three statistical models in the genome of 207 wheat varieties.

## Data Availability

The datasets used and/or analyzed during the current study are available from the corresponding author on request.

## Abbreviations

ATP: Adenosine triphosphate
BLUPs: Best linear unbiased predictors
Ca: Calcium
CTAB: Cetyltrimethylammonium bromide
FarmCPU: Fixed and random model Circulating Probability Unification
GLM: General linear models
GWAS: Genome-wide association analysis
KF: Kaifeng
MLM: Mixed linear models
PVE: Phenotypic variation explain
QQ: Quantile–quantile
QTL: Quantitative trait locus
SQ: Shangqiu
SNP: Single-nucleotide polymorphism
YY: Yuanyang
