# Supplementary material for: Genome-wide association study reveals the genetic architecture for calcium accumulation in grains of hexaploid wheat (Triticum aestivum L.)
Source: BMC Plant Biol. 2022 May 4;22:229. doi: 10.1186/s12870-022-03602-z (PMC9066855; doi:10.1186/s12870-022-03602-z)
Supplement: Supplementary file 1 — Additional file 1: Figure S1. Single nucleotide polymorphism (SNP) density (number of SNPs within 1 Mb window size) of 207 bread wheat lines analyzed with the wheat 660 K SNP assay. Figure S2. LD decay distance estimated for 207 wheat accessions. Figure S3. Histogram of the calcium concentrations in wheat grains. Data from BLUP environment are shown. Figure S4. Venn diagram of significant SNPs associated with wheat grains Ca accumulation were identified by three GLM, MLM and FarmCPU models. Figure S5. Manhattan and quantile-quantile plots for Ca concentrations using the GLM for wheat grains across different environments (including BLUP). The dashed horizontal line represents the significant threshold of −log10(P) = 4.0. The SNPs above the red dotted line are significantly associated with calcium variation. Figure S6. Manhattan and quantile-quantile plots for Ca concentrations using the MLM for wheat grains across different environments (including BLUP). The dashed horizontal line represents the significant threshold of −log10(P) = 4.0. The SNPs above the red dotted line are significantly associated with calcium variation. Figure S7. Manhattan and quantile-quantile plots for Ca concentrations using the FarmCPU for wheat grains across different environments (including BLUP). The dashed horizontal line represents the significant threshold of −log10(P) = 4.0. The SNPs above the red dotted line are significantly associated with calcium variation. Figure S8. Expression level of candidate genes in different wheat tissues. The heat map was plotted using the transcripts per kilobase million (TPM) values after log2 conversion, which were obtained from the public database of Wheat Expression Browser (http://www.wheat-expression.com). (A) the heat map of high-confidence candidate genes within 10 Mb physical intervals from the SNPs AX-110013515, (B) AX-110922471, (C) AX-94729264 and (D) AX-108912427, respectively. Table S1. Average phenotypic values of Ca accumulation in 207 whe [file 12870_2022_3602_MOESM1_ESM.docx]

**SUPPLEMENTAL INFORMATION**

**Figure S1.** Single nucleotide polymorphism (SNP) density (number of SNPs within 1Mb window size) of 207 bread wheat lines analyzed with the wheat 660K SNP assay.

**Figure S2.** LD decay distance estimated for 207 wheat accessions.

**Figure S3.** Histogram of the calcium concentrations in wheat grains. Data from BLUP environment are shown.

**Figure S4.** Venn diagram of significant SNPs associated with wheat grains Ca accumulation were identified by three GLM, MLM and FarmCPU models.

**Figure S5.** Manhattan and quantile-quantile plots for Ca concentrations using the GLM for wheat grains across different environments (including BLUP). The dashed horizontal line represents the significant threshold of −log_10_(P) = 4.0. The SNPs above the red dotted line are significantly associated with calcium variation.

**Figure S6.** Manhattan and quantile-quantile plots for Ca concentrations using the MLM for wheat grains across different environments (including BLUP). The dashed horizontal line represents the significant threshold of −log_10_(P) = 4.0. The SNPs above the red dotted line are significantly associated with calcium variation.

**Figure S7.** Manhattan and quantile-quantile plots for Ca concentrations using the FarmCPU for wheat grains across different environments (including BLUP). The dashed horizontal line represents the significant threshold of −log_10_(P) = 4.0. The SNPs above the red dotted line are significantly associated with calcium variation.

**Figure S8.** Expression level of candidate genes in different wheat tissues. The heat map was plotted using the transcripts per kilobase million (TPM) values after log_2_ conversion, which were obtained from the public database of Wheat Expression Browser (http://www.wheat-expression.com). (A) the heat map of high-confidence candidate genes within 10 Mb physical intervals from the SNPs AX-110013515, (B) AX-110922471, (C) AX-94729264 and (D) AX-108912427, respectively.

**Table S1.** Average phenotypic values of Ca accumulation in 207 wheat accessions across from each environments and BLUP.

**Table S2.** Marker-trait associations for Ca accumulation in the associated population analyzed by GLM model.

**Table S3.** Marker-trait associations for Ca accumulation in the associated population analyzed by MLM model.

**Table S4.** Marker-trait associations for Ca accumulation in the associated population analyzed by the FarmCPU model.

**Table S5.** The expression values of high-confidence candidate genes within 10 M physical intervals of the 4 stable loci identified by GWAS in different wheat tissues.

**Table S6.** Number of superior and inferior alleles across 11 significantly associated SNPs identified by three statistical models in the genome of 207 wheat varieties.


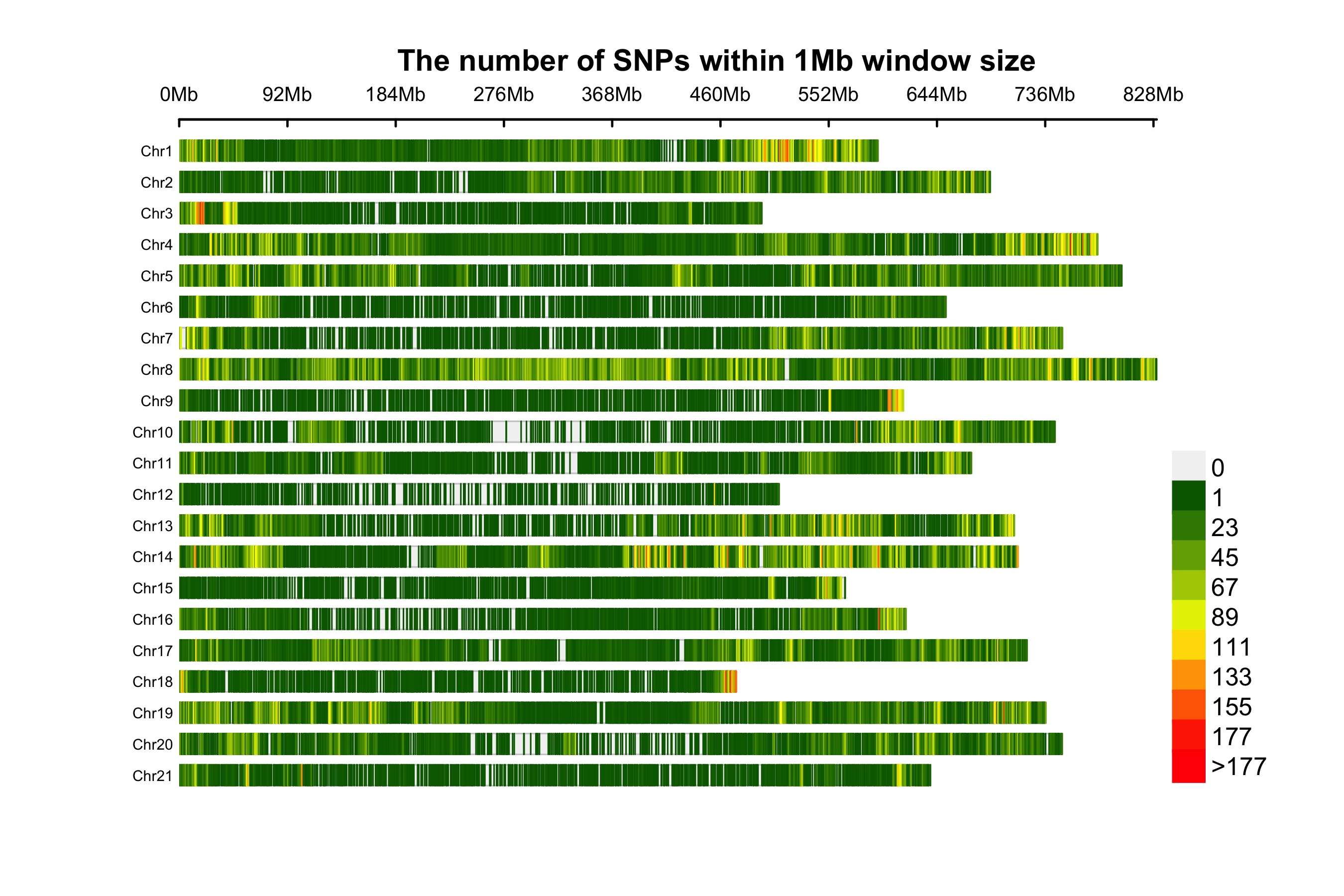
**Figure S1.** Single nucleotide polymorphism (SNP) density (number of SNPs within 1Mb window size) of 207 bread wheat lines analyzed with the wheat 660K SNP assay.

**Figure S2.** LD decay distance estimated for 207 wheat accessions.

**
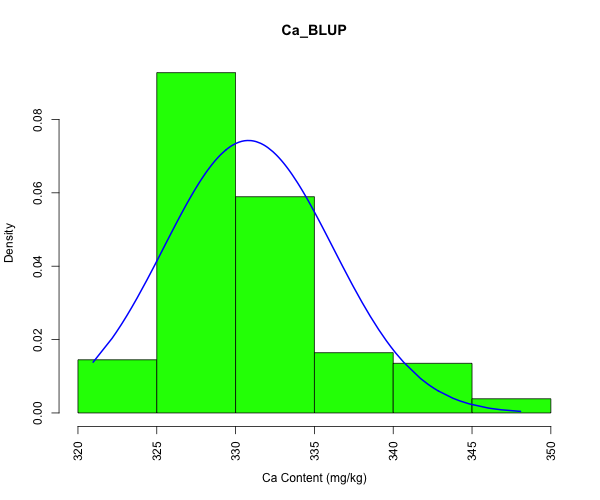
**

**Figure S3.** Histogram of the calcium concentrations in wheat grains. Data from BLUP environment are shown.


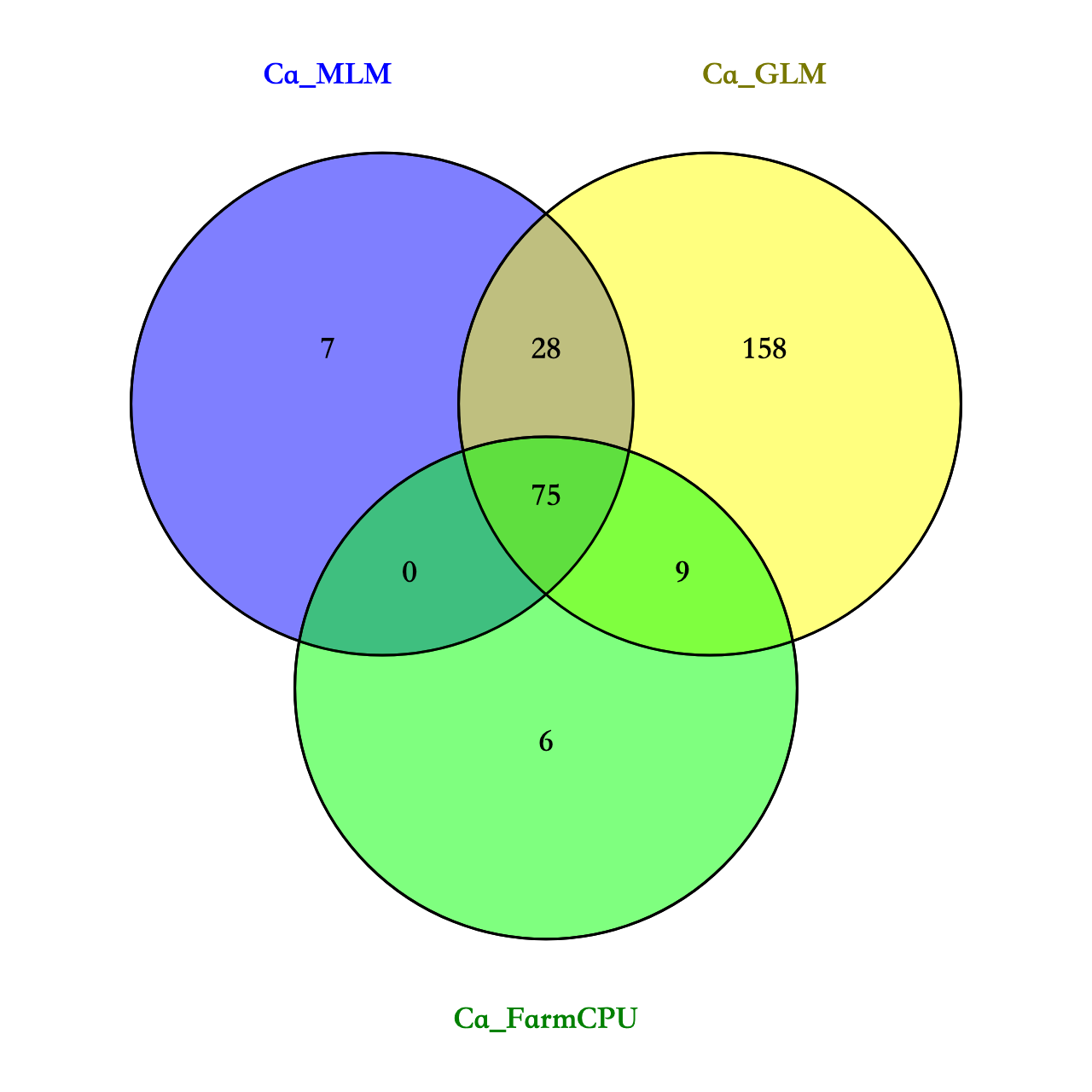


**Figure S4.** Venn diagram of significant SNPs associated with wheat grains Ca accumulation were identified by three GLM, MLM and FarmCPU models.


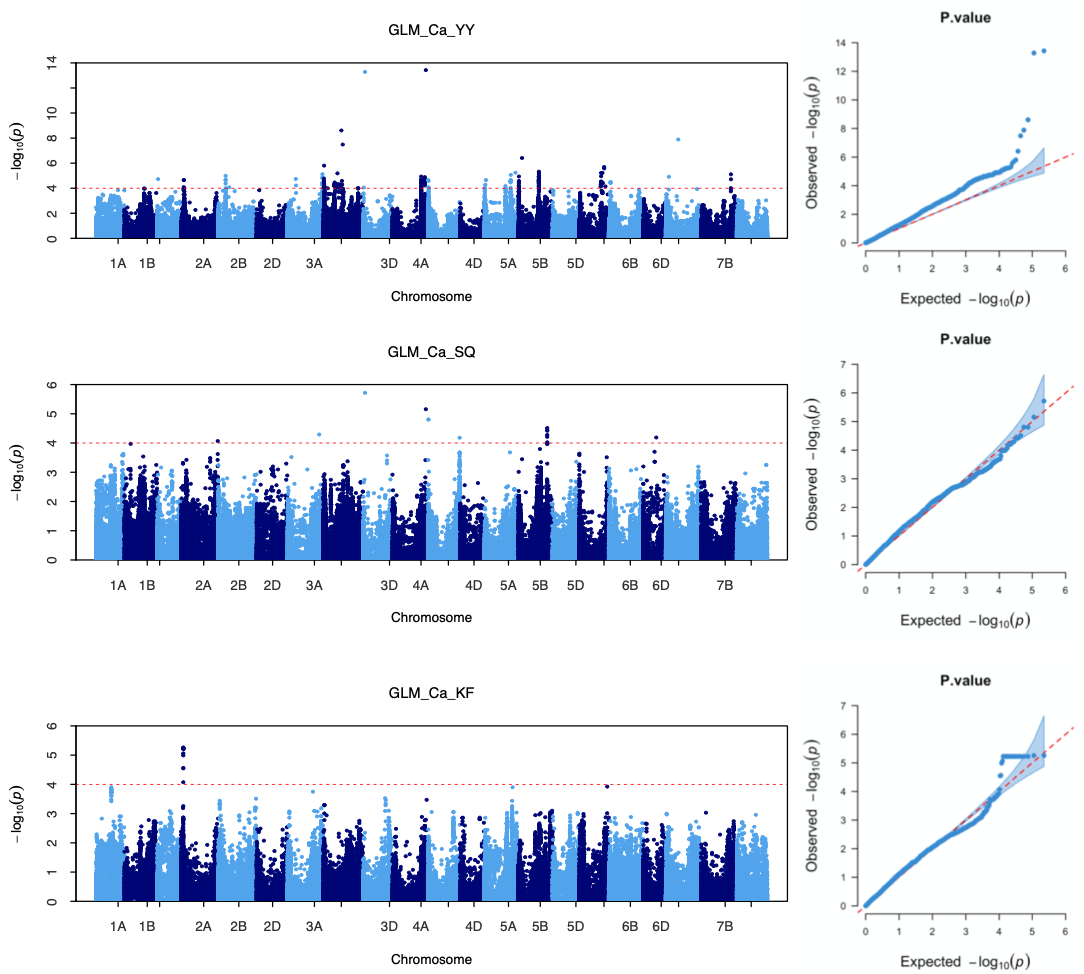


**Figure S5.** Manhattan and quantile-quantile plots for Ca concentrations using the GLM for wheat grains across different environments (including BLUP). The dashed horizontal line represents the significant threshold of −log_10_(P) = 4.0. The SNPs above the red dotted line are significantly associated with calcium variation.


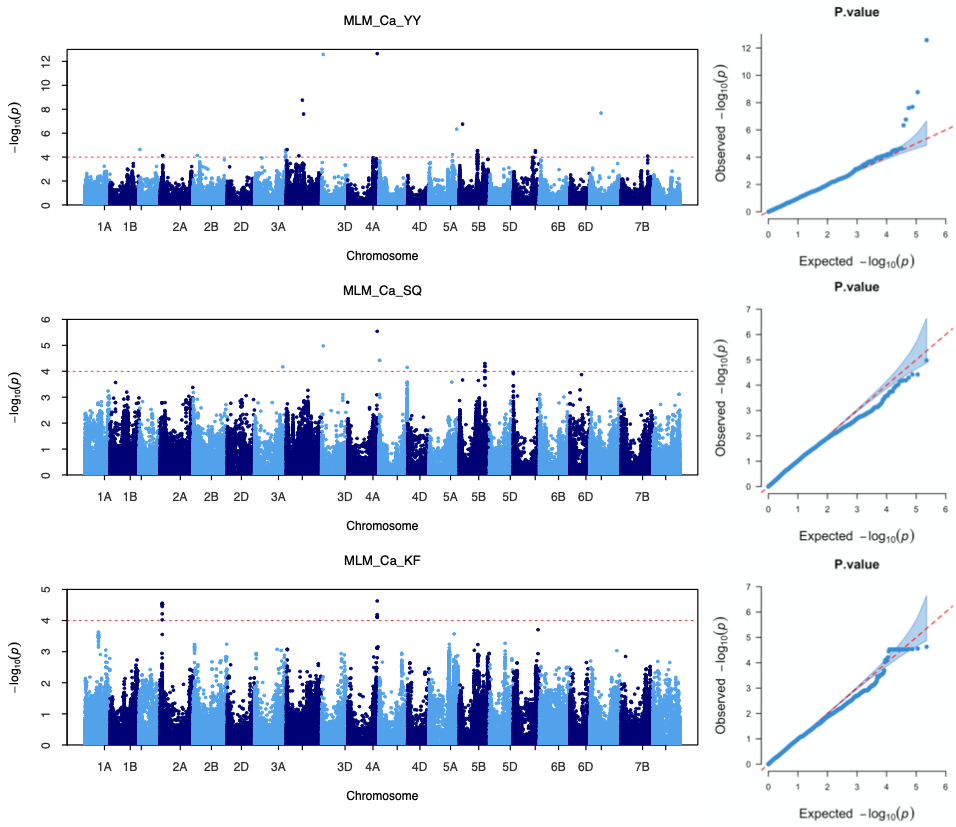


**Figure S6.** Manhattan and quantile-quantile plots for Ca concentrations using the MLM for wheat grains across different environments (including BLUP). The dashed horizontal line represents the significant threshold of −log_10_(P) = 4.0. The SNPs above the red dotted line are significantly associated with calcium variation.


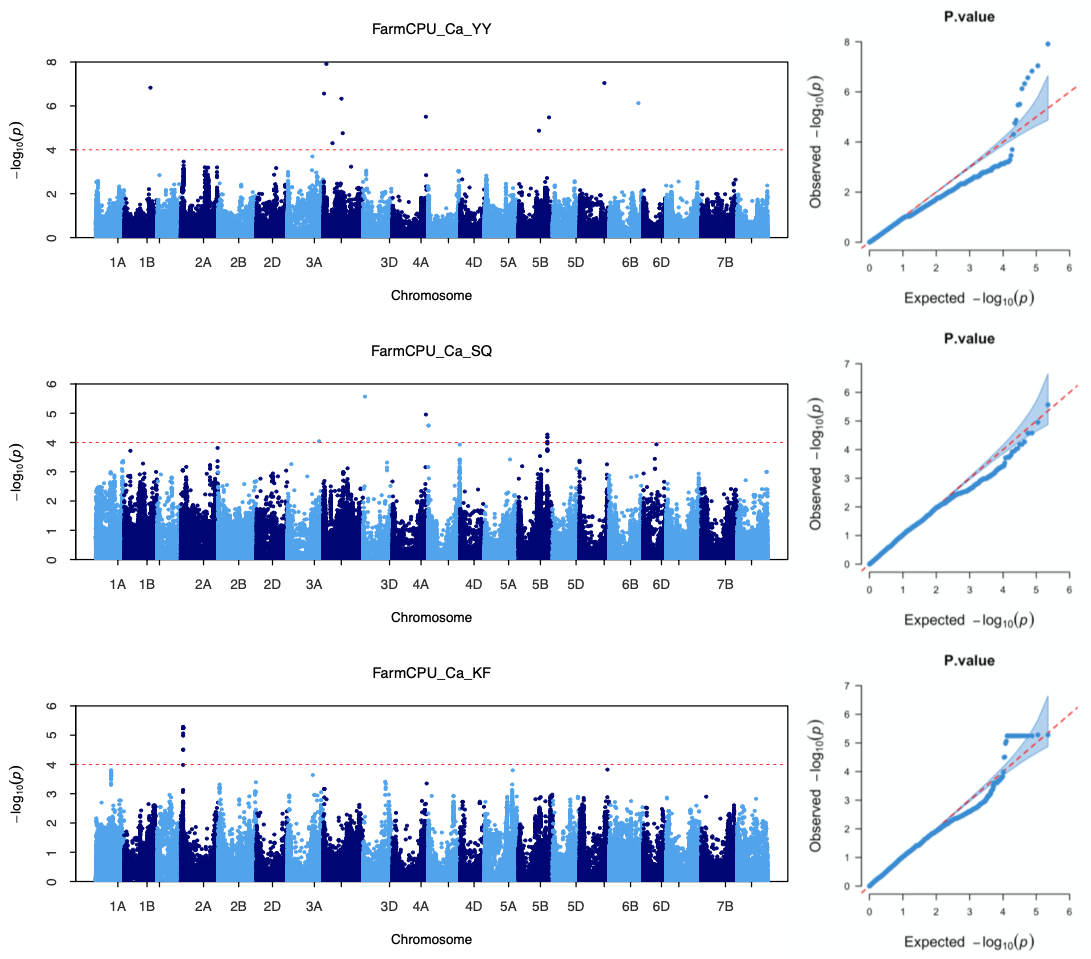


**Figure S7.** Manhattan and quantile-quantile plots for Ca concentrations using the FarmCPU for wheat grains across different environments (including BLUP). The dashed horizontal line represents the significant threshold of −log_10_(P) = 4.0. The SNPs above the red dotted line are significantly associated with calcium variation.

**Figure S8.** Expression level of candidate genes in different wheat tissues. The heat map was plotted using the transcripts per kilobase million (TPM) values after log2 conversion, which were obtained from the public database of Wheat Expression Browser (http://www.wheat-expression.com). (A) the heat map of high-confidence candidate genes within 10 Mb physical intervals from the SNPs AX-110013515, (B) AX-110922471, (C) AX-94729264 and (D) AX-108912427, respectively.

**Table S1.** Average phenotypic values of Ca accumulation in 207 wheat accessions across from each environments and BLUP.

| **Accession ID.** | **Cultivar name** | **YY** | **SQ** | **KF** | **BLUP** |
| --- | --- | --- | --- | --- | --- |
| L001 | Changwu 135 | 184.74 | 435.03 | 248.17 | 326.91 |
| L002 | Shan 229 | 239.08 | 523.39 | 304.85 | 333.15 |
| L003 | Xiaoyan 6 | 215.51 | 595.54 | 349.47 | 336.07 |
| L004 | Lantian 10 | 234.43 | 420.76 | 267.07 | 328.61 |
| L005 | Luohan 3 | 245.98 | 550 | 227.63 | 331.79 |
| L006 | Jing 411 | 180.88 | 567.16 | 225.49 | 330.22 |
| L007 | Ningdong 1 | 169.65 | 633.9 | 363.49 | 336.27 |
| L008 | Mianyang 11 | 298.89 | 486.51 | 275.45 | 332.95 |
| L009 | Taishan 5 | 332.73 | 494.2 | 269.36 | 334.06 |
| L010 | Xinmai 13 | 189.01 | 458.28 | 360.9 | 331.30 |
| L011 | Xinmai 18 | 258.49 | 546.8 | 254.89 | 332.93 |
| L012 | Zhoumai 16 | 273.18 | 558.02 | 228.34 | 332.91 |
| L013 | Yuanfeng 139 | 229.3 | 379.07 | 178.31 | 324.37 |
| L014 | Fengchan 3 | 254.32 | 376.99 | 223.69 | 326.51 |
| L015 | Zhongyu 8 | 203.33 | 395.59 | 222.57 | 325.46 |
| L016 | Bainong 160 | 217.44 | 391.9 | 202.84 | 325.17 |
| L017 | Luomai 21 | 243.67 | 404.41 | 287.35 | 329.03 |
| L018 | Yanzhan 4110 | 193.86 | 509.6 | 149.13 | 326.43 |
| L019 | Gan 6172 | 213.59 | 436.16 | 223.53 | 327.08 |
| L020 | Huaimai 21 | 220.96 | 483.78 | 124.75 | 325.71 |
| L021 | Mianyang 26 | 188.25 | 407.93 | 222.41 | 325.37 |
| L022 | Lunxuan 715 | 261.85 | 427.07 | 227.7 | 328.44 |
| L023 | Youpi 1 | 280.63 | 435.19 | 158.75 | 327.12 |
| L024 | Xifeng 9 | 228.66 | 449.14 | 140.15 | 325.35 |
| L025 | Nongda 198 | 222.57 | 380.03 | 121.71 | 322.42 |
| L026 | Kedong 81 | 200.92 | 451.87 | 228.66 | 327.34 |
| L027 | Linfeng 10 | 220.32 | 422.04 | 216.79 | 326.64 |
| L028 | Fengkang 5 | 266.53 | 395.75 | 260.89 | 328.64 |
| L029 | Changfeng 1 | 261.69 | 368.97 | 136.3 | 323.75 |
| L030 | Jingdong 1 | 187.61 | 390.62 | 314.62 | 327.69 |
| L031 | Jinmai 21 | 272.6 | 321.34 | 122.19 | 322.16 |
| L032 | Jimai 23 | 198.35 | 334.81 | 200.76 | 322.72 |
| L033 | Hanxuan 10 | 139.18 | 380.03 | 226.42 | 323.09 |
| L034 | Xianshixinmai | 153.62 | 408.9 | 174.14 | 322.81 |
| L035 | Duckbill wheat | 205.25 | 344.43 | 316.53 | 326.86 |
| L036 | Bima 1 | 182.55 | 413.74 | 258.01 | 326.49 |
| L037 | Aimengniu | 156.82 | 430.83 | 250.79 | 325.99 |
| L038 | Wenmai 6 | 170.77 | 433.29 | 222.57 | 325.62 |
| L039 | Zhameng wheat | 169.97 | 446.51 | 190.34 | 325.00 |
| L040 | Dayuhua | 172.54 | 444.11 | 191.77 | 325.05 |
| L041 | Chushanbao | 370.19 | 328.56 | 175.42 | 327.11 |
| L042 | Dalibanmang | 364.1 | 333.83 | 218.56 | 328.43 |
| L043 | Baizhameng | 162.28 | 432.37 | 238.6 | 325.83 |
| L044 | Ganmai 8 | 160.99 | 414.59 | 287.67 | 326.77 |
| L045 | Yunmai 34 | 179.59 | 383.94 | 361.11 | 328.69 |
| L046 | Wuyi wheat | 164.2 | 413.69 | 285.75 | 326.78 |
| L047 | Nanda 2419 | 201.08 | 388.13 | 317.01 | 328.11 |
| L048 | Nonglin 10 | 352.77 | 349.65 | 352.7 | 332.77 |
| L049 | Hanyang wheat | 163.56 | 362.76 | 405.45 | 328.91 |
| L050 | Lovelin 10 | 174.32 | 373.63 | 455.48 | 331.15 |
| L051 | Aifeng 3 | 167.73 | 358.58 | 471.75 | 330.99 |
| L052 | Yunhan 618 | 200.92 | 373.14 | 382.92 | 329.70 |
| L053 | Yannong 21 | 179.91 | 403.72 | 293.12 | 327.19 |
| L054 | Jinmai 47 | 207.98 | 395.01 | 278.62 | 327.34 |
| L055 | Changwu 58 | 202.68 | 373.4 | 379.71 | 329.66 |
| L056 | Zhenghan 1 | 205.57 | 386.49 | 316.85 | 328.20 |
| L057 | Chang 6878 | 215.19 | 380.03 | 330.73 | 328.73 |
| L058 | Luohan 1 | 203.65 | 394.35 | 288.63 | 327.50 |
| L059 | Luohan 6 | 229.3 | 382.39 | 294.08 | 328.10 |
| L060 | Luohan 11 | 238.44 | 382.48 | 274.29 | 327.77 |
| L061 | Puxing 5 | 214.23 | 388.82 | 292.64 | 327.78 |
| L062 | Ruiquan 24 | 180.56 | 413.69 | 261.37 | 326.53 |
| L063 | Dehongfu 2 | 168.05 | 445.59 | 195.95 | 325.09 |
| L064 | Zhongmai 895 | 195.63 | 381.13 | 353.25 | 328.86 |
| L065 | Huaimai 35 | 165.32 | 419.24 | 268.75 | 326.46 |
| L066 | Longping 518 | 200.44 | 392.4 | 301.3 | 327.73 |
| L067 | Huaimai 30 | 194.99 | 418.54 | 221.77 | 325.89 |
| L068 | Zhongyuan 6 | 202.68 | 388.42 | 313.4 | 328.06 |
| L069 | Pingan 8 | 254.64 | 379.68 | 249.51 | 327.41 |
| L070 | Bainong 207 | 207.49 | 378.08 | 351.33 | 329.07 |
| L071 | Cun 1 | 221.45 | 395.66 | 250.31 | 326.90 |
| L072 | Zhoumai 26 | 206.21 | 289.59 | 322.31 | 325.36 |
| L073 | Luomai 18 | 217.44 | 320.38 | 452.61 | 330.75 |
| L074 | Baofeng 10-82 | 215.99 | 363.04 | 367.78 | 329.38 |
| L075 | Su 553 | 226.9 | 326.15 | 329.62 | 327.38 |
| L076 | 09N37 | 230.91 | 385.32 | 402.1 | 331.62 |
| L077 | Guinong 17 | 194.67 | 304.03 | 381.57 | 327.30 |
| L078 | Hengguan 35 | 199.64 | 303.71 | 417.33 | 328.57 |
| L079 | Yumai 18 | 209.42 | 314.93 | 455.5 | 330.42 |
| L080 | Yumai 13 | 242.13 | 387.41 | 418.78 | 332.56 |
| L081 | Yumai 47 | 274.68 | 389.01 | 410.12 | 333.36 |
| L082 | Zhengmai 004 | 232.83 | 382.92 | 438.02 | 332.73 |
| L083 | Yumai 50 | 241.17 | 391.58 | NA | 329.51 |
| L084 | Taikong 6 | 214.87 | 391.26 | 378.05 | 330.55 |
| L085 | Huapei 5 | 307.55 | 365.6 | 325.85 | 331.02 |
| L086 | Hua 9987 | 192.42 | 335.78 | 450.53 | 330.38 |
| L087 | Yujiao 5 | 267.15 | 358.87 | 391.03 | 331.58 |
| L088 | Yunong 416 | 240.37 | 368.49 | 433.85 | 332.38 |
| L089 | Bainong 64 | 345.72 | 396.07 | 463.19 | 337.46 |
| L090 | Zhoumai 9 | 417.23 | 417.23 | 388.63 | 338.03 |
| L091 | Zhoumai 13 | 306.27 | 424.29 | 323.21 | 332.73 |
| L092 | Zhoumai 8425B | 194.99 | 399.6 | 371.31 | 329.98 |
| L093 | Kaimai 21 | 156.18 | 412.58 | 303.78 | 327.06 |
| L094 | Luozhen 1 | 159.87 | 416.74 | 487.09 | 333.04 |
| L095 | Neixiang 188 | 247.26 | 404.89 | 440.9 | 333.96 |
| L096 | Aiyou 26-2 | 196.27 | 457.48 | 437.94 | 333.92 |
| L097 | Changgeheimai | 194.03 | 435.35 | 355.92 | 330.59 |
| L098 | Lvmai 1 | 173.18 | 347.96 | 351.75 | 327.07 |
| L099 | Subeimai 1 | 230.1 | 456.52 | 433.21 | 334.80 |
| L100 | Jinan 17 | 676.04 | 415.95 | 386.38 | 346.02 |
| L101 | Jimai 20 | 661.61 | 406.65 | 372.27 | 344.83 |
| L102 | Shan 225 | 335.29 | 445.94 | 393.76 | 336.52 |
| L103 | Xinong 979 | 545.68 | 442.09 | 354.31 | 341.75 |
| L104 | Baxter | 513.28 | 443.37 | 335.07 | 340.18 |
| L105 | CD87 | 585.92 | 454.28 | 321.6 | 342.37 |
| L106 | Kukri | 564.44 | 403.12 | 375.96 | 341.80 |
| L107 | Faguomai | 430.86 | 438.08 | 273.9 | 335.52 |
| L108 | Belero | 539.26 | 254.89 | 317.11 | 334.53 |
| L109 | Fundulea 900 | 538.46 | 276.21 | 327.86 | 335.51 |
| L110 | Tincurrin | 510.88 | 226.26 | 286.33 | 331.78 |
| L111 | H149 | 634.35 | 330.59 | 291.78 | 339.08 |
| L112 | Fa B08 | 378.75 | 271.34 | 317.43 | 330.03 |
| L113 | Fa B20 | 537.98 | 309.72 | 302.84 | 335.76 |
| L114 | Bainong 416 | 448.34 | 258.24 | 296.27 | 331.14 |
| L115 | Luo 31 | 449.63 | 345.82 | 339.88 | 335.28 |
| L116 | Taihemai 1 | 477.21 | 314.75 | 310.7 | 334.26 |
| L117 | Xunmai 35 | 507.99 | 220.47 | 300.44 | 331.95 |
| L118 | Luomai 23 | 599.39 | 338.51 | 343.41 | 339.85 |
| L119 | Jiaomai 266 | 422.69 | 372.78 | 329.3 | 334.95 |
| L120 | Wennong 14 | 464.38 | 303.48 | 303.93 | 333.29 |
| L121 | Jimai 22 | 501.26 | 344.14 | 356.56 | 337.37 |
| L122 | Ningmai 9 | 358.87 | 358.61 | 314.55 | 332.05 |
| L123 | 10EW28 | 487.79 | 392.88 | 460.47 | 341.72 |
| L124 | 10EW137 | 545.68 | 282 | 411.88 | 338.54 |
| L125 | Zhou 18 | 486.02 | 378.26 | 423.27 | 340.05 |
| L126 | 04Z38 | 539.42 | 492.65 | 375.16 | 343.79 |
| L127 | Yumai 34-9901 | 571.17 | 363.18 | 440.74 | 342.78 |
| L128 | Zheng 9023-8 | 353.73 | 382.37 | 367.46 | 334.29 |
| L129 | Zheng 103 | 459.25 | 415.27 | 433.21 | 340.68 |
| L130 | Calingiri | 559.63 | 436.9 | 487.73 | 346.20 |
| L131 | Zhengmai 366 | 498.21 | 370.04 | 487.73 | 342.19 |
| L132 | Zhengmai 7698 | 284.46 | 342.47 | 483.24 | 334.49 |
| L133 | Zhoumai 22 | 319.1 | 358.46 | 506.01 | 336.79 |
| L134 | Zhoumai 27 | 488.11 | 541.08 | 514.99 | 348.08 |
| L135 | Zhoumai 30 | 405.37 | 483.51 | 489.01 | 342.87 |
| L136 | Zhoumai 32 | 372.34 | 580.53 | 552.19 | 346.85 |
| L137 | Liangxing 66 | 347.32 | 504.07 | 518.67 | 342.63 |
| L138 | Emai 25 | 439.52 | 447.87 | 533.59 | 344.22 |
| L139 | Ningchun5 | 336.26 | 397.76 | 291.14 | 331.83 |
| L140 | Shannong 33 | 374.1 | 455.79 | 274.46 | 334.31 |
| L141 | Zhongmai 175 | 403.92 | 332.11 | 261.95 | 330.98 |
| L142 | Gaocheng 8901 | 286.07 | 279.56 | 255.22 | 325.44 |
| L143 | Shannongtedali 1 | 310.76 | 406.74 | 247.84 | 329.96 |
| L144 | Changmai 9 | 355.02 | 447.41 | 268.21 | 333.26 |
| L145 | Yangfumai 2 | 334.17 | 405.68 | 269.01 | 331.32 |
| L146 | Wanmai 53 | 415.31 | 413.9 | 247.2 | 333.44 |
| L147 | Fanmai 8 | 313.65 | 476.05 | 236.62 | 331.87 |
| L148 | Yunmai 51 | 407.93 | 383.29 | 259.87 | 332.65 |
| L149 | Yunmai 53 | 240.92 | 356.18 | 246.56 | 326.16 |
| L150 | Yunmai 47 | 292.81 | 382.68 | 251.05 | 328.75 |
| L151 | Xinong 928 | 226.41 | 343.99 | 292.42 | 326.75 |
| L152 | Xinmai 26 | 371.71 | 293.88 | 246.88 | 328.31 |
| L153 | Wanmai 47 | 260.53 | 384.96 | 273.83 | 328.52 |
| L154 | Yangmai 13 | 320.08 | 321.3 | 278.95 | 328.55 |
| L155 | Gaoyou 2018 | 319.16 | 414.05 | 320.64 | 332.73 |
| L156 | Gaoyou 9415 | 247.56 | 276.82 | 276.22 | 324.81 |
| L157 | Shiluan 02-1 | 256.41 | 420.91 | 271.09 | 329.43 |
| L158 | Zheng 1005 | 252.76 | 260.22 | 307.49 | 325.43 |
| L159 | Zheng 1105 | 231.28 | 350.39 | 308.77 | 327.62 |
| L160 | Zheng 1118 | 247.12 | 612.06 | 275.26 | 335.25 |
| L161 | Zheng 1289 | 296.86 | 331.96 | 329.94 | 329.76 |
| L162 | Zheng 3093 | 250.02 | 429.13 | 276.86 | 329.67 |
| L163 | Sanyuehuang | 208.13 | 298.6 | 248.8 | 323.40 |
| L164 | Wuhuatou | 225.08 | 390.45 | 291.78 | 328.15 |
| L165 | Tutoumai | 202.8 | 381.46 | 311.82 | 327.80 |
| L166 | Baisuibai | 236.16 | 289.11 | 314.23 | 326.02 |
| L167 | Qumangmai | 282 | 685.17 | 304.93 | 339.56 |
| L168 | Zhengzhou 6 | 278.8 | 207.37 | 300.12 | 324.36 |
| L169 | Zhengyin 1 | 257.63 | 303.63 | 290.82 | 326.42 |
| L170 | Shengxuan 7 | 325.26 | 309.26 | 286.65 | 328.58 |
| L171 | Shuangfengshou | 317.79 | 409.64 | 279.75 | 331.27 |
| L172 | Zhengzhou 7 | 254.28 | 267.99 | 292.42 | 325.25 |
| L173 | Wanchangsui | 211.11 | 311.09 | 278.63 | 324.81 |
| L174 | Yuanzhu | 408.11 | 366.38 | 306.66 | 333.59 |
| L175 | Longfumai 4 | 263.05 | 284.74 | 332.03 | 327.29 |
| L176 | Yizheng 8165 | 280.02 | 296.62 | 278.79 | 326.52 |
| L177 | Taihan 2 | 303.17 | 270.07 | 294.34 | 326.90 |
| L178 | Neixiang 182 | 250.02 | 381.61 | 263.07 | 327.75 |
| L179 | Chuanmai 50 | 294.95 | 205.7 | 297.34 | 324.73 |
| L180 | Chuanmai 46 | 184.37 | 189.09 | 304.28 | 320.96 |
| L181 | Chuanmai 44 | 269.21 | 253.52 | 320 | 326.13 |
| L182 | Chuanmai 107 | 334.55 | 304.54 | 287.93 | 328.76 |
| L183 | Jinyang 35 | 270.73 | 243.01 | 302.36 | 325.29 |
| L184 | Zhengmai 101 | 225.8 | 264.33 | 308.77 | 324.75 |
| L185 | Zhengmai 518 | 254.58 | 290.99 | 344.37 | 327.60 |
| L186 | AK 58 | 308.05 | 255.65 | 294.34 | 326.61 |
| L187 | Shannong 22 | 279.11 | 218.94 | 313.91 | 325.16 |
| L188 | Nongda 211 | 358 | 220.92 | 342.65 | 328.59 |
| L189 | Shannong 19 | 255.19 | 339.57 | 354.31 | 329.45 |
| L190 | Shannong 06-278 | 258.7 | 273.93 | 272.86 | 324.96 |
| L191 | Yangmai 18 | 309.32 | 281.04 | 356.4 | 329.38 |
| L192 | Ningmai 17 | 381.32 | 220.31 | 395.69 | 330.96 |
| L193 | Ningmai 13 | 446.1 | 262.96 | 383.66 | 333.95 |
| L194 | Zhenmai 168 | 393.82 | 187.87 | 384.46 | 329.99 |
| L195 | Yangmai 12 | 452.19 | 264.42 | 442.83 | 336.04 |
| L196 | Yangmai 14 | 425.57 | 288.09 | 387.51 | 334.21 |
| L197 | Huamai 5 | 398.31 | 343.53 | 367.78 | 334.48 |
| L198 | Yangmai 20 | 338.34 | 380.85 | 394.88 | 334.62 |
| L199 | Yangmai 21 | 288.31 | 411.16 | 366.18 | 333.10 |
| L200 | Zhengmai 379 | 367.14 | 269.66 | 341.81 | 330.38 |
| L201 | Zhengmai 05706 | 287.03 | 218.49 | 362.33 | 326.91 |
| L202 | Zhengmai 113 | 331.5 | 346.73 | 363.61 | 332.36 |
| L203 | Zhengmai 369 | 289.92 | 471.93 | 338.28 | 334.18 |
| L204 | Zhengmai 05871 | 327.08 | 237.22 | 342.13 | 328.12 |
| L205 | Xinmai 19 | 300.58 | 355.26 | 396.97 | 332.70 |
| L206 | Shannong 26 | 394.56 | 261.13 | 329.14 | 330.57 |
| L207 | Shijiazhuang 8 | 212.14 | 305.61 | 379.17 | 327.82 |

YY, SQ, and KF are Yuanyang 2017, Shangqiu 2017 and Kaifeng 2017 in Yellow and Huai wheat region, respectively.

The best linear unbiased predictions (BLUP) values calculated from all three environments averaged phenotypic values.

**Table S2.** Marker-trait associations for Ca accumulation in the associated population analyzed by GLM model.

| **Environment^1^** | **SNP marker^2^** | **Chromosome** | **Physical position(bp)** | **P.value^3^** | **R^2^(%)^4^** |
| --- | --- | --- | --- | --- | --- |
| YY | AX-110529533 | 1D | 16132987 | 1.88E-05 | 14.06 |
| YY | AX-109868359 | 2A | 58461459 | 8.23E-05 | 12.63 |
| YY | AX-111076209 | 2A | 59067345 | 2.24E-05 | 13.89 |
| YY | AX-111644808 | 2A | 59067408 | 2.24E-05 | 13.89 |
| YY | AX-111504996 | 2A | 59127575 | 9.74E-05 | 12.46 |
| YY | AX-94723778 | 2B | 154911185 | 2.46E-05 | 13.80 |
| YY | AX-94509448 | 2B | 154911296 | 2.07E-05 | 13.97 |
| YY | AX-94609661 | 2B | 154911419 | 4.32E-05 | 13.25 |
| YY | AX-110620516 | 2B | 154930484 | 1.03E-05 | 14.65 |
| YY | AX-109904133 | 2B | 229992624 | 8.45E-05 | 12.60 |
| YY | AX-109994498 | 3A | 171184072 | 1.83E-05 | 14.09 |
| YY | AX-94712805 | 3A | 176558184 | 6.07E-05 | 12.92 |
| YY | AX-111656976 | 3A | 728515342 | 4.87E-05 | 13.13 |
| YY | AX-108754885 | 3A | 731006056 | 3.81E-05 | 13.37 |
| YY | AX-111575165 | 3A | 731007209 | 1.29E-05 | 14.44 |
| YY | AX-108856079 | 3A | 731008208 | 5.46E-05 | 13.02 |
| YY | AX-109399066 | 3A | 731068214 | 7.53E-06 | 14.97 |
| YY | AX-109375863 | 3A | 731084608 | 1.61E-05 | 14.21 |
| YY | AX-99469873 | 3A | 731086521 | 3.76E-05 | 13.39 |
| YY | AX-89587299 | 3A | 731086768 | 3.76E-05 | 13.39 |
| YY | AX-111722901 | 3B | 10304342 | 3.95E-05 | 13.34 |
| YY | AX-89363542 | 3B | 11087167 | 1.69E-05 | 14.17 |
| YY | AX-94951301 | 3B | 11087168 | 2.82E-05 | 13.67 |
| YY | AX-94865981 | 3B | 11087211 | 7.45E-05 | 12.72 |
| YY | AX-94867446 | 3B | 11087250 | 2.69E-05 | 13.71 |
| YY | AX-110013515 | 3B | 13967085 | 1.58E-06 | 16.54 |
| YY | AX-109955117 | 3B | 17088232 | 4.66E-05 | 13.18 |
| YY | AX-108737500 | 3B | 21169796 | 7.53E-05 | 12.71 |
| YY | AX-110673512 | 3B | 21337886 | 7.53E-05 | 12.71 |
| YY | AX-108942431 | 3B | 224839218 | 6.19E-05 | 12.90 |
| YY | AX-111258884 | 3B | 224932429 | 6.07E-05 | 12.92 |
| YY | AX-109578788 | 3B | 225450317 | 6.07E-05 | 12.92 |
| YY | AX-111455047 | 3B | 225463713 | 6.07E-05 | 12.92 |
| YY | AX-109410691 | 3B | 226003241 | 6.07E-05 | 12.92 |
| YY | AX-112287376 | 3B | 227418801 | 6.07E-05 | 12.92 |
| YY | AX-109956526 | 3B | 227439694 | 6.07E-05 | 12.92 |
| YY | AX-111032082 | 3B | 227666060 | 6.19E-05 | 12.90 |
| YY | AX-109907005 | 3B | 228088387 | 6.07E-05 | 12.92 |
| YY | AX-108957628 | 3B | 228089841 | 6.07E-05 | 12.92 |
| YY | AX-108790932 | 3B | 228098657 | 4.71E-05 | 13.17 |
| YY | AX-108750022 | 3B | 228269027 | 5.27E-05 | 13.06 |
| YY | AX-110648424 | 3B | 228749110 | 6.07E-05 | 12.92 |
| YY | AX-109561642 | 3B | 229006732 | 6.07E-05 | 12.92 |
| YY | AX-109610399 | 3B | 234916170 | 6.07E-05 | 12.92 |
| YY | AX-111552638 | 3B | 235916784 | 6.07E-05 | 12.92 |
| YY | AX-109530923 | 3B | 236149565 | 6.07E-05 | 12.92 |
| YY | AX-111008432 | 3B | 236365413 | 3.44E-05 | 13.47 |
| YY | AX-109891636 | 3B | 236822402 | 6.07E-05 | 12.92 |
| YY | AX-110094824 | 3B | 254844514 | 9.14E-05 | 12.53 |
| YY | AX-109363602 | 3B | 280897825 | 8.69E-05 | 12.57 |
| YY | AX-110675149 | 3B | 282236586 | 5.06E-05 | 13.10 |
| YY | AX-110015709 | 3B | 287176245 | 5.06E-05 | 13.10 |
| YY | AX-110201772 | 3B | 294521180 | 6.51E-06 | 15.11 |
| YY | AX-110545181 | 3B | 296203673 | 5.06E-05 | 13.10 |
| YY | AX-111527975 | 3B | 313308894 | 5.06E-05 | 13.10 |
| YY | AX-108921911 | 3B | 331336286 | 9.93E-05 | 12.45 |
| YY | AX-110559693 | 3B | 339076592 | 5.06E-05 | 13.10 |
| YY | AX-108868525 | 3B | 347797877 | 5.06E-05 | 13.10 |
| YY | AX-108760150 | 3B | 364542782 | 8.84E-05 | 12.56 |
| YY | AX-110922471 | 3B | 376625452 | 2.46E-09 | 23.37 |
| YY | AX-110676983 | 3B | 380733584 | 5.79E-05 | 12.97 |
| YY | AX-110582504 | 3B | 387980933 | 2.61E-05 | 13.74 |
| YY | AX-110690027 | 3B | 391787133 | 5.06E-05 | 13.10 |
| YY | AX-109359598 | 3B | 404801032 | 3.27E-08 | 20.58 |
| YY | AX-94974843 | 3B | 727323211 | 9.55E-05 | 12.48 |
| YY | AX-111041089 | 3D | 15924112 | 8.83E-05 | 12.56 |
| YY | AX-94729264 | 3D | 40526440 | 5.25E-10 | 25.87 |
| YY | AX-109930726 | 4A | 601179123 | 3.71E-05 | 13.40 |
| YY | AX-111479635 | 4A | 601372300 | 5.47E-05 | 13.02 |
| YY | AX-110380791 | 4A | 602060630 | 8.27E-05 | 12.62 |
| YY | AX-109425179 | 4A | 602083536 | 3.10E-05 | 13.57 |
| YY | AX-109966767 | 4A | 602099739 | 8.27E-05 | 12.62 |
| YY | AX-109843777 | 4A | 602173445 | 2.19E-05 | 13.92 |
| YY | AX-109327618 | 4A | 602176945 | 2.05E-05 | 13.98 |
| YY | AX-110482680 | 4A | 602179163 | 1.16E-05 | 14.54 |
| YY | AX-111134927 | 4A | 602184728 | 2.19E-05 | 13.92 |
| YY | AX-110507419 | 4A | 602241447 | 2.54E-05 | 13.77 |
| YY | AX-89661808 | 4A | 602258030 | 8.27E-05 | 12.62 |
| YY | AX-111551635 | 4A | 602258226 | 8.27E-05 | 12.62 |
| YY | AX-109953368 | 4A | 604014829 | 3.92E-05 | 13.35 |
| YY | AX-111825287 | 4A | 676781724 | 1.30E-05 | 14.43 |
| YY | AX-111498824 | 4A | 676852517 | 3.43E-05 | 13.47 |
| YY | AX-111613933 | 4A | 676909168 | 1.83E-05 | 14.09 |
| YY | AX-108973418 | 4A | 677119034 | 2.28E-05 | 13.88 |
| YY | AX-109374586 | 4A | 677564160 | 2.38E-05 | 13.83 |
| YY | AX-111701433 | 4A | 677657058 | 3.43E-05 | 13.47 |
| YY | AX-110485149 | 4A | 677673132 | 3.43E-05 | 13.47 |
| YY | AX-109496641 | 4A | 677810444 | 1.80E-05 | 14.10 |
| YY | AX-110975877 | 4A | 677863768 | 1.80E-05 | 14.10 |
| YY | AX-108911364 | 4A | 677935831 | 6.03E-05 | 12.93 |
| YY | AX-110412137 | 4A | 677935995 | 1.59E-05 | 14.23 |
| YY | AX-109894155 | 4A | 677966538 | 3.43E-05 | 13.47 |
| YY | AX-109877087 | 4A | 677971879 | 1.80E-05 | 14.10 |
| YY | AX-111079487 | 4A | 677980260 | 1.80E-05 | 14.10 |
| YY | AX-111697651 | 4A | 677994641 | 1.80E-05 | 14.10 |
| YY | AX-111511829 | 4A | 678040517 | 1.83E-05 | 14.09 |
| YY | AX-110547493 | 4A | 678049494 | 3.86E-05 | 13.36 |
| YY | AX-110122648 | 4A | 678053941 | 1.83E-05 | 14.09 |
| YY | AX-108876876 | 4A | 678078267 | 3.43E-05 | 13.47 |
| YY | AX-111592047 | 4A | 678079302 | 4.53E-05 | 13.20 |
| YY | AX-108912427 | 4A | 699571654 | 3.73E-14 | 36.29 |
| YY | AX-95156879 | 4B | 3861096 | 2.19E-05 | 13.92 |
| YY | AX-111464226 | 4B | 10708472 | 9.21E-05 | 12.52 |
| YY | AX-108846551 | 4B | 11965665 | 8.43E-05 | 12.60 |
| YY | AX-109074448 | 4B | 12116835 | 2.62E-05 | 13.74 |
| YY | AX-110977568 | 5A | 18691581 | 5.30E-05 | 13.05 |
| YY | AX-109416721 | 5A | 31419022 | 2.23E-05 | 13.89 |
| YY | AX-110504347 | 5A | 441188443 | 6.71E-05 | 12.82 |
| YY | AX-110038187 | 5A | 547591836 | 3.52E-05 | 13.45 |
| YY | AX-110916805 | 5A | 547629281 | 2.84E-05 | 13.66 |
| YY | AX-111657611 | 5A | 548287244 | 2.73E-05 | 13.70 |
| YY | AX-94674693 | 5A | 548626291 | 2.73E-05 | 13.70 |
| YY | AX-109585440 | 5A | 549142489 | 9.35E-05 | 12.50 |
| YY | AX-89487067 | 5A | 549161554 | 2.73E-05 | 13.70 |
| YY | AX-109850781 | 5A | 552279888 | 2.18E-05 | 13.92 |
| YY | AX-110435122 | 5A | 552755844 | 8.70E-06 | 14.83 |
| YY | AX-110931014 | 5A | 650240330 | 5.79E-06 | 15.23 |
| YY | AX-108733795 | 5B | 10453057 | 6.25E-05 | 12.89 |
| YY | AX-109983278 | 5B | 10576776 | 6.25E-05 | 12.89 |
| YY | AX-109363472 | 5B | 13099776 | 3.72E-05 | 13.39 |
| YY | AX-110914174 | 5B | 13101195 | 2.77E-05 | 13.68 |
| YY | AX-111706457 | 5B | 13167540 | 3.00E-05 | 13.60 |
| YY | AX-111561716 | 5B | 13168172 | 1.16E-05 | 14.54 |
| YY | AX-111603302 | 5B | 13169146 | 2.74E-05 | 13.69 |
| YY | AX-110377739 | 5B | 13170065 | 3.72E-05 | 13.39 |
| YY | AX-109502121 | 5B | 13363266 | 7.79E-05 | 12.68 |
| YY | AX-112289745 | 5B | 78708064 | 3.88E-07 | 17.98 |
| YY | AX-109449556 | 5B | 431072335 | 2.28E-05 | 13.87 |
| YY | AX-111719873 | 5B | 431073840 | 3.01E-05 | 13.60 |
| YY | AX-110941511 | 5B | 431074967 | 1.23E-05 | 14.48 |
| YY | AX-110436085 | 5B | 431075394 | 7.83E-06 | 14.93 |
| YY | AX-108753683 | 5B | 431075720 | 4.77E-06 | 15.42 |
| YY | AX-109906166 | 5B | 431076319 | 2.86E-05 | 13.65 |
| YY | AX-109917916 | 5B | 431079242 | 2.28E-05 | 13.87 |
| YY | AX-109347631 | 5B | 431081256 | 2.83E-05 | 13.66 |
| YY | AX-108939963 | 5B | 431084695 | 4.63E-05 | 13.18 |
| YY | AX-108891185 | 5B | 431094139 | 4.55E-05 | 13.20 |
| YY | AX-108977887 | 5B | 431263397 | 2.28E-05 | 13.87 |
| YY | AX-108753210 | 5B | 431347007 | 1.23E-05 | 14.48 |
| YY | AX-108792770 | 5B | 431350837 | 1.23E-05 | 14.48 |
| YY | AX-110555831 | 5B | 431351040 | 2.92E-05 | 13.63 |
| YY | AX-108737248 | 5B | 431352441 | 5.55E-06 | 15.27 |
| YY | AX-108796489 | 5B | 431352697 | 2.17E-05 | 13.92 |
| YY | AX-111035174 | 5B | 431352931 | 3.00E-05 | 13.60 |
| YY | AX-110995399 | 5B | 431353031 | 1.85E-05 | 14.08 |
| YY | AX-109395201 | 5B | 431359284 | 2.28E-05 | 13.87 |
| YY | AX-111577616 | 5B | 431359554 | 1.23E-05 | 14.48 |
| YY | AX-109838027 | 5B | 431360545 | 2.28E-05 | 13.87 |
| YY | AX-110561849 | 5B | 431364382 | 1.39E-05 | 14.36 |
| YY | AX-111057239 | 5B | 431383087 | 2.28E-05 | 13.87 |
| YY | AX-110140657 | 5B | 431383813 | 1.23E-05 | 14.48 |
| YY | AX-111591290 | 5B | 431394134 | 3.07E-05 | 13.58 |
| YY | AX-111826177 | 5B | 431401090 | 2.28E-05 | 13.87 |
| YY | AX-110546869 | 5B | 431401791 | 2.67E-05 | 13.72 |
| YY | AX-108810511 | 5B | 431611904 | 7.05E-05 | 12.78 |
| YY | AX-108873416 | 5B | 431711473 | 1.74E-05 | 14.14 |
| YY | AX-110417925 | 5B | 431713239 | 4.63E-05 | 13.18 |
| YY | AX-109858007 | 5B | 431734637 | 4.38E-05 | 13.24 |
| YY | AX-110672081 | 5B | 431735985 | 1.97E-05 | 14.02 |
| YY | AX-108850578 | 5B | 431869008 | 9.82E-05 | 12.46 |
| YY | AX-111151537 | 5B | 431869830 | 4.24E-05 | 13.27 |
| YY | AX-111517584 | 5B | 431870293 | 4.24E-05 | 13.27 |
| YY | AX-111455485 | 5B | 431870921 | 3.28E-05 | 13.52 |
| YY | AX-110453589 | 5B | 431877684 | 4.56E-05 | 13.20 |
| YY | AX-111257124 | 5B | 431892280 | 6.73E-05 | 12.82 |
| YY | AX-111083587 | 5B | 431936879 | 6.73E-05 | 12.82 |
| YY | AX-109580736 | 5B | 431937206 | 6.42E-05 | 12.87 |
| YY | AX-110670730 | 5B | 431979358 | 6.73E-05 | 12.82 |
| YY | AX-111060640 | 5B | 432004945 | 8.76E-05 | 12.57 |
| YY | AX-109993885 | 5B | 432005009 | 3.75E-05 | 13.39 |
| YY | AX-110045668 | 5B | 432005238 | 6.73E-05 | 12.82 |
| YY | AX-109645590 | 5B | 432005436 | 6.73E-05 | 12.82 |
| YY | AX-111013588 | 5B | 432055235 | 3.75E-05 | 13.39 |
| YY | AX-110125864 | 5B | 432110024 | 3.40E-05 | 13.48 |
| YY | AX-109391958 | 5B | 432110374 | 6.73E-05 | 12.82 |
| YY | AX-109312806 | 5B | 432126317 | 6.73E-05 | 12.82 |
| YY | AX-111534635 | 5B | 432126654 | 3.75E-05 | 13.39 |
| YY | AX-110510229 | 6A | 447632581 | 7.11E-05 | 12.77 |
| YY | AX-108870168 | 6A | 448663617 | 7.11E-05 | 12.77 |
| YY | AX-110383372 | 6A | 449359124 | 4.92E-05 | 13.12 |
| YY | AX-108896824 | 6A | 452269985 | 5.33E-05 | 13.05 |
| YY | AX-111457927 | 6A | 455942645 | 4.16E-05 | 13.29 |
| YY | AX-111276079 | 6A | 455946110 | 4.16E-05 | 13.29 |
| YY | AX-110436089 | 6A | 455986930 | 2.98E-05 | 13.61 |
| YY | AX-108767470 | 6A | 455991636 | 4.16E-05 | 13.29 |
| YY | AX-111034463 | 6A | 455991714 | 3.03E-05 | 13.59 |
| YY | AX-108958326 | 6A | 456330273 | 4.16E-05 | 13.29 |
| YY | AX-111471212 | 6A | 456504345 | 4.16E-05 | 13.29 |
| YY | AX-108774814 | 6A | 456601479 | 4.16E-05 | 13.29 |
| YY | AX-111007569 | 6A | 456605791 | 3.03E-05 | 13.59 |
| YY | AX-109433081 | 6A | 456762342 | 6.03E-06 | 15.19 |
| YY | AX-108947389 | 6A | 456783069 | 1.09E-05 | 14.60 |
| YY | AX-110620101 | 6A | 456783303 | 5.74E-05 | 12.97 |
| YY | AX-108838621 | 6A | 483549102 | 6.03E-06 | 15.19 |
| YY | AX-108770374 | 6A | 483587688 | 3.50E-05 | 13.45 |
| YY | AX-111106634 | 6A | 490486081 | 4.06E-05 | 13.31 |
| YY | AX-111186262 | 6A | 520871113 | 2.64E-06 | 16.02 |
| YY | AX-111183340 | 6A | 520873173 | 2.03E-06 | 16.29 |
| YY | AX-110428239 | 6A | 546479778 | 7.20E-05 | 12.76 |
| YY | AX-110516679 | 6B | 34928573 | 3.16E-05 | 13.55 |
| YY | AX-109922037 | 6B | 35001108 | 3.46E-05 | 13.47 |
| YY | AX-109933860 | 6B | 35228399 | 3.78E-05 | 13.38 |
| YY | AX-111071918 | 6B | 35229143 | 3.78E-05 | 13.38 |
| YY | AX-109085312 | 7A | 63756956 | 1.22E-05 | 14.49 |
| YY | AX-111012263 | 7A | 261687749 | 1.30E-08 | 21.57 |
| YY | AX-94571186 | 7B | 626068901 | 9.89E-05 | 12.45 |
| YY | AX-111148246 | 7B | 626954936 | 7.74E-06 | 14.94 |
| YY | AX-95140513 | 7B | 627982714 | 1.94E-05 | 14.03 |
| SQ | AX-111099812 | 2A | 770023298 | 8.56E-05 | 10.09 |
| SQ | AX-109541359 | 3A | 662398903 | 5.11E-05 | 10.61 |
| SQ | AX-94729264 | 3D | 40526440 | 1.92E-06 | 13.96 |
| SQ | AX-108912427 | 4A | 699571654 | 6.97E-06 | 12.62 |
| SQ | AX-110637930 | 4B | 10703582 | 1.57E-05 | 11.79 |
| SQ | AX-108994873 | 4B | 10703651 | 1.57E-05 | 11.79 |
| SQ | AX-110646581 | 4B | 663903130 | 6.61E-05 | 10.35 |
| SQ | AX-111141605 | 5B | 606299294 | 3.08E-05 | 11.11 |
| SQ | AX-108986427 | 5B | 606301590 | 3.75E-05 | 10.91 |
| SQ | AX-110992849 | 5B | 606304982 | 6.15E-05 | 10.42 |
| SQ | AX-111452566 | 5B | 606306921 | 5.30E-05 | 10.57 |
| SQ | AX-111626317 | 5B | 606468177 | 9.43E-05 | 10.00 |
| SQ | AX-111573332 | 5B | 606814678 | 3.75E-05 | 10.91 |
| SQ | AX-111137903 | 6D | 272644054 | 6.48E-05 | 10.37 |
| KF | AX-109874999 | 2A | 49069207 | 5.52E-06 | 18.15 |
| KF | AX-110505263 | 2A | 49074491 | 5.96E-06 | 18.07 |
| KF | AX-111503867 | 2A | 49075226 | 5.96E-06 | 18.07 |
| KF | AX-110547163 | 2A | 49163115 | 5.96E-06 | 18.07 |
| KF | AX-110128660 | 2A | 49165325 | 5.96E-06 | 18.07 |
| KF | AX-108901218 | 2A | 49165822 | 5.96E-06 | 18.07 |
| KF | AX-111058921 | 2A | 49165881 | 5.96E-06 | 18.07 |
| KF | AX-110439302 | 2A | 49166746 | 8.44E-05 | 15.55 |
| KF | AX-111507490 | 2A | 49167020 | 5.96E-06 | 18.07 |
| KF | AX-110386322 | 2A | 49168353 | 5.96E-06 | 18.07 |
| KF | AX-111717596 | 2A | 49168884 | 5.96E-06 | 18.07 |
| KF | AX-108785477 | 2A | 49170016 | 5.96E-06 | 18.07 |
| KF | AX-109015046 | 2A | 49170760 | 5.96E-06 | 18.07 |
| KF | AX-109921295 | 2A | 49174588 | 2.74E-05 | 16.61 |
| KF | AX-109986057 | 2A | 49174939 | 8.70E-06 | 17.71 |
| KF | AX-109415725 | 2A | 49176324 | 5.96E-06 | 18.07 |
| KF | AX-110496107 | 2A | 49177395 | 2.84E-05 | 16.57 |
| KF | AX-109950804 | 2A | 49178351 | 1.02E-05 | 17.55 |
| KF | AX-111145879 | 2A | 49181270 | 5.96E-06 | 18.07 |
| KF | AX-110519744 | 2A | 49182908 | 5.96E-06 | 18.07 |
| KF | AX-109426178 | 2A | 49183814 | 5.96E-06 | 18.07 |
| KF | AX-110634514 | 2A | 49219472 | 5.54E-06 | 18.14 |
| BLUP | AX-111799835 | 3A | 38138188 | 2.47E-05 | 18.65 |
| BLUP | AX-109455393 | 3A | 38141372 | 2.47E-05 | 18.65 |
| BLUP | AX-109499374 | 3B | 2151413 | 7.43E-05 | 17.64 |
| BLUP | AX-110082523 | 3B | 17960521 | 6.69E-05 | 17.73 |
| BLUP | AX-110533377 | 3B | 17994378 | 9.77E-05 | 17.39 |
| BLUP | AX-111574519 | 3B | 17996250 | 6.90E-05 | 17.70 |
| BLUP | AX-108968311 | 3B | 20469603 | 8.77E-05 | 17.49 |
| BLUP | AX-108773846 | 3B | 20478542 | 2.99E-05 | 18.47 |
| BLUP | AX-109973512 | 3B | 20478891 | 2.99E-05 | 18.47 |
| BLUP | AX-108817856 | 3B | 20482654 | 2.99E-05 | 18.47 |
| BLUP | AX-108929308 | 3B | 20483944 | 2.99E-05 | 18.47 |
| BLUP | AX-109399456 | 3B | 20504151 | 4.32E-05 | 18.13 |
| BLUP | AX-110922471 | 3B | 376625452 | 3.38E-05 | 18.36 |
| BLUP | AX-94729264 | 3D | 40526440 | 8.19E-06 | 19.68 |
| BLUP | AX-111825287 | 4A | 676781724 | 2.10E-05 | 18.80 |
| BLUP | AX-111498824 | 4A | 676852517 | 3.32E-05 | 18.38 |
| BLUP | AX-111613933 | 4A | 676909168 | 3.56E-05 | 18.31 |
| BLUP | AX-108973418 | 4A | 677119034 | 4.60E-05 | 18.07 |
| BLUP | AX-109374586 | 4A | 677564160 | 2.63E-05 | 18.59 |
| BLUP | AX-111701433 | 4A | 677657058 | 3.32E-05 | 18.38 |
| BLUP | AX-110485149 | 4A | 677673132 | 3.32E-05 | 18.38 |
| BLUP | AX-109496641 | 4A | 677810444 | 3.17E-05 | 18.42 |
| BLUP | AX-110975877 | 4A | 677863768 | 3.17E-05 | 18.42 |
| BLUP | AX-108911364 | 4A | 677935831 | 5.04E-05 | 17.99 |
| BLUP | AX-110412137 | 4A | 677935995 | 1.96E-05 | 18.86 |
| BLUP | AX-109894155 | 4A | 677966538 | 3.32E-05 | 18.38 |
| BLUP | AX-109877087 | 4A | 677971879 | 3.17E-05 | 18.42 |
| BLUP | AX-111079487 | 4A | 677980260 | 3.17E-05 | 18.42 |
| BLUP | AX-111697651 | 4A | 677994641 | 3.17E-05 | 18.42 |
| BLUP | AX-111511829 | 4A | 678040517 | 3.56E-05 | 18.31 |
| BLUP | AX-110547493 | 4A | 678049494 | 6.45E-05 | 17.77 |
| BLUP | AX-110122648 | 4A | 678053941 | 3.56E-05 | 18.31 |
| BLUP | AX-108876876 | 4A | 678078267 | 3.32E-05 | 18.38 |
| BLUP | AX-111592047 | 4A | 678079302 | 2.54E-05 | 18.62 |
| BLUP | AX-109978529 | 4A | 678130103 | 2.99E-05 | 18.47 |
| BLUP | AX-108966947 | 4A | 678135098 | 2.39E-05 | 18.68 |
| BLUP | AX-110466232 | 4A | 678135759 | 2.70E-05 | 18.56 |
| BLUP | AX-111228468 | 4A | 678178441 | 2.70E-05 | 18.56 |
| BLUP | AX-109337003 | 4A | 678178783 | 2.99E-05 | 18.47 |
| BLUP | AX-110131192 | 4A | 678201265 | 2.70E-05 | 18.56 |
| BLUP | AX-109416575 | 4A | 678214031 | 2.99E-05 | 18.47 |
| BLUP | AX-110475236 | 4A | 678214928 | 2.99E-05 | 18.47 |
| BLUP | AX-110627066 | 4A | 678215097 | 2.99E-05 | 18.47 |
| BLUP | AX-110625914 | 4A | 678215738 | 2.70E-05 | 18.56 |
| BLUP | AX-110589063 | 4A | 678216569 | 2.70E-05 | 18.56 |
| BLUP | AX-110554623 | 4A | 678219137 | 2.70E-05 | 18.56 |
| BLUP | AX-110127878 | 4A | 678260692 | 4.02E-05 | 18.20 |
| BLUP | AX-110543806 | 4A | 678856863 | 2.99E-05 | 18.47 |
| BLUP | AX-108912427 | 4A | 699571654 | 1.75E-06 | 21.14 |
| BLUP | AX-111462163 | 5B | 10450732 | 4.03E-05 | 18.20 |
| BLUP | AX-108993073 | 5B | 10451609 | 3.98E-05 | 18.21 |
| BLUP | AX-110914174 | 5B | 13101195 | 9.71E-05 | 17.39 |
| BLUP | AX-111561716 | 5B | 13168172 | 7.25E-05 | 17.66 |
| BLUP | AX-108986501 | 5B | 13168628 | 6.23E-05 | 17.80 |
| BLUP | AX-111603302 | 5B | 13169146 | 9.62E-05 | 17.40 |
| BLUP | AX-109085312 | 7A | 63756956 | 7.12E-05 | 17.68 |

^1^ Phenotypic values collected from three locations (KF, 2017 Kaifeng; SQ, 2017 Shangqiu; YY, 2017 Yuanyang), and the best linear unbiased predictions (BLUP) values calculated from all three environments.

^2^ Markers were detected at the threshold -log_10_(P) equaling 4.0.

^3^ *P-value* of the corresponding significant SNPs calculated by GLM model.

^4^ R^2^ of Model with significant SNP.

**Table S3.** Marker-trait associations for Ca accumulation in the associated population analyzed by MLM model.

| **Environment^1^** | **SNP marker^2^** | **Chromosome** | **Physical position(bp)** | **P-value^3^** | **R^2^ (%)^4^** |
| --- | --- | --- | --- | --- | --- |
| YY | AX-110529533 | 1D | 16132987 | 2.30E-05 | 15.04 |
| YY | AX-110634514 | 2A | 49219472 | 7.01E-05 | 13.89 |
| YY | AX-111644808 | 2A | 59067408 | 7.61E-05 | 13.89 |
| YY | AX-110970921 | 2B | 95738751 | 7.21E-05 | 13.95 |
| YY | AX-109541359 | 3A | 662398903 | 1.77E-05 | 14.91 |
| YY | AX-108754885 | 3A | 731006056 | 6.75E-05 | 14.01 |
| YY | AX-111575165 | 3A | 731007209 | 2.68E-05 | 14.89 |
| YY | AX-109399066 | 3A | 731068214 | 3.54E-05 | 14.62 |
| YY | AX-109375863 | 3A | 731084608 | 3.15E-05 | 14.74 |
| YY | AX-99469873 | 3A | 731086521 | 8.35E-05 | 13.81 |
| YY | AX-89587299 | 3A | 731086768 | 8.35E-05 | 13.81 |
| YY | AX-110013515 | 3B | 13967085 | 2.33E-05 | 15.03 |
| YY | AX-110201772 | 3B | 294521180 | 7.63E-05 | 13.89 |
| YY | AX-110922471 | 3B | 376625452 | 1.71E-09 | 24.81 |
| YY | AX-109359598 | 3B | 404801032 | 2.47E-08 | 21.95 |
| YY | AX-94729264 | 3D | 40526440 | 2.65E-13 | 26.80 |
| YY | AX-108912427 | 4A | 699571654 | 2.24E-13 | 26.93 |
| YY | AX-110435122 | 5A | 552755844 | 6.18E-05 | 14.09 |
| YY | AX-110931014 | 5A | 650240330 | 4.61E-07 | 18.92 |
| YY | AX-112289745 | 5B | 78708064 | 1.74E-07 | 19.92 |
| YY | AX-110941511 | 5B | 431074967 | 5.91E-05 | 14.13 |
| YY | AX-110436085 | 5B | 431075394 | 3.17E-05 | 14.73 |
| YY | AX-108753683 | 5B | 431075720 | 8.71E-05 | 13.77 |
| YY | AX-108753210 | 5B | 431347007 | 5.91E-05 | 14.13 |
| YY | AX-108792770 | 5B | 431350837 | 5.91E-05 | 14.13 |
| YY | AX-108737248 | 5B | 431352441 | 2.84E-05 | 14.84 |
| YY | AX-110995399 | 5B | 431353031 | 8.22E-05 | 13.82 |
| YY | AX-111577616 | 5B | 431359554 | 5.91E-05 | 14.13 |
| YY | AX-110561849 | 5B | 431364382 | 6.59E-05 | 14.03 |
| YY | AX-110140657 | 5B | 431383813 | 5.91E-05 | 14.13 |
| YY | AX-110546869 | 5B | 431401791 | 8.93E-05 | 13.74 |
| YY | AX-108873416 | 5B | 431711473 | 7.89E-05 | 13.86 |
| YY | AX-110672081 | 5B | 431735985 | 8.74E-05 | 13.76 |
| YY | AX-111186262 | 6A | 520871113 | 3.91E-05 | 14.53 |
| YY | AX-111183340 | 6A | 520873173 | 2.88E-05 | 14.83 |
| YY | AX-111012263 | 7A | 261687749 | 2.06E-08 | 22.15 |
| YY | AX-111148246 | 7B | 626954936 | 8.24E-05 | 13.82 |
| SQ | AX-109541359 | 3A | 662398903 | 6.77E-05 | 9.66 |
| SQ | AX-94729264 | 3D | 40526440 | 1.05E-05 | 11.55 |
| SQ | AX-108912427 | 4A | 699571654 | 2.87E-06 | 12.89 |
| SQ | AX-110637930 | 4B | 10703582 | 3.79E-05 | 10.24 |
| SQ | AX-108994873 | 4B | 10703651 | 3.79E-05 | 10.24 |
| SQ | AX-110646581 | 4B | 663903130 | 7.10E-05 | 9.61 |
| SQ | AX-111141605 | 5B | 606299294 | 5.00E-05 | 9.96 |
| SQ | AX-108986427 | 5B | 606301590 | 6.45E-05 | 9.71 |
| SQ | AX-111452566 | 5B | 606306921 | 9.38E-05 | 9.33 |
| SQ | AX-111573332 | 5B | 606814678 | 6.45E-05 | 9.71 |
| KF | AX-109874999 | 2A | 49069207 | 2.97E-05 | 16.09 |
| KF | AX-110505263 | 2A | 49074491 | 2.99E-05 | 16.08 |
| KF | AX-111503867 | 2A | 49075226 | 2.99E-05 | 16.08 |
| KF | AX-110547163 | 2A | 49163115 | 2.99E-05 | 16.08 |
| KF | AX-110128660 | 2A | 49165325 | 2.99E-05 | 16.08 |
| KF | AX-108901218 | 2A | 49165822 | 2.99E-05 | 16.08 |
| KF | AX-111058921 | 2A | 49165881 | 2.99E-05 | 16.08 |
| KF | AX-111507490 | 2A | 49167020 | 2.99E-05 | 16.08 |
| KF | AX-110386322 | 2A | 49168353 | 2.99E-05 | 16.08 |
| KF | AX-111717596 | 2A | 49168884 | 2.99E-05 | 16.08 |
| KF | AX-108785477 | 2A | 49170016 | 2.99E-05 | 16.08 |
| KF | AX-109015046 | 2A | 49170760 | 2.99E-05 | 16.08 |
| KF | AX-109921295 | 2A | 49174588 | 6.11E-05 | 15.01 |
| KF | AX-109986057 | 2A | 49174939 | 2.91E-05 | 16.09 |
| KF | AX-109415725 | 2A | 49176324 | 2.91E-05 | 16.09 |
| KF | AX-109950804 | 2A | 49178351 | 3.56E-05 | 15.53 |
| KF | AX-111145879 | 2A | 49181270 | 2.99E-05 | 16.08 |
| KF | AX-110519744 | 2A | 49182908 | 2.99E-05 | 16.08 |
| KF | AX-109426178 | 2A | 49183814 | 2.99E-05 | 16.08 |
| KF | AX-110634514 | 2A | 49219472 | 2.76E-05 | 16.10 |
| KF | AX-109453201 | 4A | 698905259 | 7.94E-05 | 7.32 |
| KF | AX-108826914 | 4A | 698906030 | 6.46E-05 | 7.37 |
| KF | AX-110946462 | 4A | 698906848 | 7.60E-05 | 7.32 |
| KF | AX-111077932 | 4A | 699101825 | 7.60E-05 | 7.32 |
| KF | AX-111607567 | 4A | 699570974 | 7.60E-05 | 7.32 |
| KF | AX-108912427 | 4A | 699571654 | 2.35E-05 | 16.55 |
| BLUP | AX-111799835 | 3A | 38138188 | 5.27E-05 | 17.09 |
| BLUP | AX-109455393 | 3A | 38141372 | 5.27E-05 | 17.09 |
| BLUP | AX-109499374 | 3B | 2151413 | 9.18E-05 | 16.57 |
| BLUP | AX-110013515 | 3B | 13967085 | 3.33E-05 | 18.03 |
| BLUP | AX-108847344 | 3B | 14103942 | 3.95E-05 | 17.64 |
| BLUP | AX-108773846 | 3B | 20478542 | 6.21E-05 | 16.93 |
| BLUP | AX-109973512 | 3B | 20478891 | 6.21E-05 | 16.93 |
| BLUP | AX-108817856 | 3B | 20482654 | 6.21E-05 | 16.93 |
| BLUP | AX-108929308 | 3B | 20483944 | 6.21E-05 | 16.93 |
| BLUP | AX-109399456 | 3B | 20504151 | 9.96E-05 | 16.50 |
| BLUP | AX-110922471 | 3B | 376625452 | 5.56E-05 | 17.04 |
| BLUP | AX-94729264 | 3D | 40526440 | 1.68E-05 | 18.15 |
| BLUP | AX-111825287 | 4A | 676781724 | 4.14E-05 | 17.31 |
| BLUP | AX-111498824 | 4A | 676852517 | 6.50E-05 | 16.89 |
| BLUP | AX-111613933 | 4A | 676909168 | 6.96E-05 | 16.83 |
| BLUP | AX-108973418 | 4A | 677119034 | 9.40E-05 | 16.55 |
| BLUP | AX-109374586 | 4A | 677564160 | 5.22E-05 | 17.09 |
| BLUP | AX-111701433 | 4A | 677657058 | 6.50E-05 | 16.89 |
| BLUP | AX-110485149 | 4A | 677673132 | 6.50E-05 | 16.89 |
| BLUP | AX-109496641 | 4A | 677810444 | 6.30E-05 | 16.92 |
| BLUP | AX-110975877 | 4A | 677863768 | 6.30E-05 | 16.92 |
| BLUP | AX-108911364 | 4A | 677935831 | 9.40E-05 | 16.55 |
| BLUP | AX-110412137 | 4A | 677935995 | 4.19E-05 | 17.30 |
| BLUP | AX-109894155 | 4A | 677966538 | 6.50E-05 | 16.89 |
| BLUP | AX-109877087 | 4A | 677971879 | 6.30E-05 | 16.92 |
| BLUP | AX-111079487 | 4A | 677980260 | 6.30E-05 | 16.92 |
| BLUP | AX-111697651 | 4A | 677994641 | 6.30E-05 | 16.92 |
| BLUP | AX-111511829 | 4A | 678040517 | 6.96E-05 | 16.83 |
| BLUP | AX-110122648 | 4A | 678053941 | 6.96E-05 | 16.83 |
| BLUP | AX-108876876 | 4A | 678078267 | 6.50E-05 | 16.89 |
| BLUP | AX-111592047 | 4A | 678079302 | 5.59E-05 | 17.03 |
| BLUP | AX-109978529 | 4A | 678130103 | 6.25E-05 | 16.93 |
| BLUP | AX-108966947 | 4A | 678135098 | 5.60E-05 | 17.03 |
| BLUP | AX-110466232 | 4A | 678135759 | 5.70E-05 | 17.01 |
| BLUP | AX-111228468 | 4A | 678178441 | 5.70E-05 | 17.01 |
| BLUP | AX-109337003 | 4A | 678178783 | 6.25E-05 | 16.93 |
| BLUP | AX-110131192 | 4A | 678201265 | 5.70E-05 | 17.01 |
| BLUP | AX-109416575 | 4A | 678214031 | 6.25E-05 | 16.93 |
| BLUP | AX-110475236 | 4A | 678214928 | 6.25E-05 | 16.93 |
| BLUP | AX-110627066 | 4A | 678215097 | 6.25E-05 | 16.93 |
| BLUP | AX-110625914 | 4A | 678215738 | 5.70E-05 | 17.01 |
| BLUP | AX-110589063 | 4A | 678216569 | 5.70E-05 | 17.01 |
| BLUP | AX-110554623 | 4A | 678219137 | 5.70E-05 | 17.01 |
| BLUP | AX-110127878 | 4A | 678260692 | 9.92E-05 | 16.50 |
| BLUP | AX-110543806 | 4A | 678856863 | 6.25E-05 | 16.93 |
| BLUP | AX-108912427 | 4A | 699571654 | 3.83E-06 | 19.55 |

^1^ Phenotypic values collected from three locations (KF, 2017 Kaifeng; SQ, 2017 Shangqiu; YY, 2017 Yuanyang), and the best linear unbiased predictions (BLUP) values calculated from all three environments.

^2^ Markers were detected at the threshold -log_10_(P) equaling 4.0.

^3^ *P-value* of the corresponding significant SNPs calculated by MLM model.

^4^ R^2^ of Model with significant SNP.

**Table S4.** Marker-trait associations for Ca accumulation in the associated population analyzed by the FarmCPU model.

| **Environment^1^** | **SNP marker^2^** | **Chromosome** | **Physical position(bp)** | **P.value^3^** |
| --- | --- | --- | --- | --- |
| YY | AX-108948156 | 1B | 549417777 | 1.48E-07 |
| YY | AX-110013515 | 3B | 13967085 | 2.74E-07 |
| YY | AX-111577061 | 3B | 64309606 | 1.22E-08 |
| YY | AX-111503468 | 3B | 191188054 | 4.97E-05 |
| YY | AX-110676434 | 3B | 379009609 | 4.71E-07 |
| YY | AX-109359598 | 3B | 404801032 | 1.75E-05 |
| YY | AX-108912427 | 4A | 699571654 | 3.11E-06 |
| YY | AX-108753683 | 5B | 431075720 | 1.35E-05 |
| YY | AX-110396257 | 5B | 640398834 | 3.34E-06 |
| YY | AX-111183340 | 6A | 520873173 | 9.07E-08 |
| YY | AX-108855608 | 6B | 615098607 | 7.45E-07 |
| SQ | AX-109541359 | 3A | 662398903 | 9.04E-05 |
| SQ | AX-94729264 | 3D | 40526440 | 2.71E-06 |
| SQ | AX-108912427 | 4A | 699571654 | 1.11E-05 |
| SQ | AX-110637930 | 4B | 10703582 | 2.64E-05 |
| SQ | AX-108994873 | 4B | 10703651 | 2.64E-05 |
| SQ | AX-111141605 | 5B | 606299294 | 5.34E-05 |
| SQ | AX-108986427 | 5B | 606301590 | 6.57E-05 |
| SQ | AX-111452566 | 5B | 606306921 | 9.39E-05 |
| SQ | AX-111573332 | 5B | 606814678 | 6.57E-05 |
| KF | AX-109874999 | 2A | 49069207 | 5.21E-06 |
| KF | AX-110505263 | 2A | 49074491 | 5.67E-06 |
| KF | AX-111503867 | 2A | 49075226 | 5.67E-06 |
| KF | AX-110547163 | 2A | 49163115 | 5.67E-06 |
| KF | AX-110128660 | 2A | 49165325 | 5.67E-06 |
| KF | AX-108901218 | 2A | 49165822 | 5.67E-06 |
| KF | AX-111058921 | 2A | 49165881 | 5.67E-06 |
| KF | AX-111507490 | 2A | 49167020 | 5.67E-06 |
| KF | AX-110386322 | 2A | 49168353 | 5.67E-06 |
| KF | AX-111717596 | 2A | 49168884 | 5.67E-06 |
| KF | AX-108785477 | 2A | 49170016 | 5.67E-06 |
| KF | AX-109015046 | 2A | 49170760 | 5.67E-06 |
| KF | AX-109921295 | 2A | 49174588 | 3.09E-05 |
| KF | AX-109986057 | 2A | 49174939 | 8.68E-06 |
| KF | AX-109415725 | 2A | 49176324 | 5.67E-06 |
| KF | AX-110496107 | 2A | 49177395 | 3.20E-05 |
| KF | AX-109950804 | 2A | 49178351 | 1.04E-05 |
| KF | AX-111145879 | 2A | 49181270 | 5.67E-06 |
| KF | AX-110519744 | 2A | 49182908 | 5.67E-06 |
| KF | AX-109426178 | 2A | 49183814 | 5.67E-06 |
| KF | AX-110634514 | 2A | 49219472 | 5.23E-06 |
| BLUP | AX-111799835 | 3A | 38138188 | 2.93E-05 |
| BLUP | AX-109455393 | 3A | 38141372 | 2.93E-05 |
| BLUP | AX-109499374 | 3B | 2151413 | 9.58E-05 |
| BLUP | AX-110082523 | 3B | 17960521 | 8.58E-05 |
| BLUP | AX-111574519 | 3B | 17996250 | 8.86E-05 |
| BLUP | AX-108773846 | 3B | 20478542 | 3.61E-05 |
| BLUP | AX-109973512 | 3B | 20478891 | 3.61E-05 |
| BLUP | AX-108817856 | 3B | 20482654 | 3.61E-05 |
| BLUP | AX-108929308 | 3B | 20483944 | 3.61E-05 |
| BLUP | AX-109399456 | 3B | 20504151 | 5.37E-05 |
| BLUP | AX-110922471 | 3B | 376625452 | 4.12E-05 |
| BLUP | AX-94729264 | 3D | 40526440 | 8.73E-06 |
| BLUP | AX-111825287 | 4A | 676781724 | 2.46E-05 |
| BLUP | AX-111498824 | 4A | 676852517 | 4.04E-05 |
| BLUP | AX-111613933 | 4A | 676909168 | 4.36E-05 |
| BLUP | AX-108973418 | 4A | 677119034 | 5.75E-05 |
| BLUP | AX-109374586 | 4A | 677564160 | 3.15E-05 |
| BLUP | AX-111701433 | 4A | 677657058 | 4.04E-05 |
| BLUP | AX-110485149 | 4A | 677673132 | 4.04E-05 |
| BLUP | AX-109496641 | 4A | 677810444 | 3.85E-05 |
| BLUP | AX-110975877 | 4A | 677863768 | 3.85E-05 |
| BLUP | AX-108911364 | 4A | 677935831 | 6.34E-05 |
| BLUP | AX-110412137 | 4A | 677935995 | 2.29E-05 |
| BLUP | AX-109894155 | 4A | 677966538 | 4.04E-05 |
| BLUP | AX-109877087 | 4A | 677971879 | 3.85E-05 |
| BLUP | AX-111079487 | 4A | 677980260 | 3.85E-05 |
| BLUP | AX-111697651 | 4A | 677994641 | 3.85E-05 |
| BLUP | AX-111511829 | 4A | 678040517 | 4.36E-05 |
| BLUP | AX-110547493 | 4A | 678049494 | 8.25E-05 |
| BLUP | AX-110122648 | 4A | 678053941 | 4.36E-05 |
| BLUP | AX-108876876 | 4A | 678078267 | 4.04E-05 |
| BLUP | AX-111592047 | 4A | 678079302 | 3.02E-05 |
| BLUP | AX-109978529 | 4A | 678130103 | 3.61E-05 |
| BLUP | AX-108966947 | 4A | 678135098 | 2.84E-05 |
| BLUP | AX-110466232 | 4A | 678135759 | 3.24E-05 |
| BLUP | AX-111228468 | 4A | 678178441 | 3.24E-05 |
| BLUP | AX-109337003 | 4A | 678178783 | 3.61E-05 |
| BLUP | AX-110131192 | 4A | 678201265 | 3.24E-05 |
| BLUP | AX-109416575 | 4A | 678214031 | 3.61E-05 |
| BLUP | AX-110475236 | 4A | 678214928 | 3.61E-05 |
| BLUP | AX-110627066 | 4A | 678215097 | 3.61E-05 |
| BLUP | AX-110625914 | 4A | 678215738 | 3.24E-05 |
| BLUP | AX-110589063 | 4A | 678216569 | 3.24E-05 |
| BLUP | AX-110554623 | 4A | 678219137 | 3.24E-05 |
| BLUP | AX-110127878 | 4A | 678260692 | 4.98E-05 |
| BLUP | AX-110543806 | 4A | 678856863 | 3.61E-05 |
| BLUP | AX-108912427 | 4A | 699571654 | 1.52E-06 |
| BLUP | AX-111462163 | 5B | 10450732 | 4.98E-05 |
| BLUP | AX-108993073 | 5B | 10451609 | 4.92E-05 |
| BLUP | AX-111561716 | 5B | 13168172 | 9.33E-05 |
| BLUP | AX-108986501 | 5B | 13168628 | 7.95E-05 |
| BLUP | AX-109085312 | 7A | 63756956 | 9.16E-05 |

^1^ Phenotypic values collected from three locations (KF, 2017 Kaifeng; SQ, 2017 Shangqiu; YY, 2017 Yuanyang), and the best linear unbiased predictions (BLUP) values calculated from all three environments.

^2^ Markers were detected at the threshold -log_10_(P) equaling 4.0.

^3^ *P-value* of the corresponding significant SNPs calculated by FarmCPU model.

**Table S5.** The expression values of high-confidence candidate genes within 10 M physical intervals of the 4 stable loci identified by GWAS in different wheat tissues.

| **Peak SNPs** | **Gene accession number** | **Roots** | **Leaves/Shoots** | **Spike** | **Grain** |
| --- | --- | --- | --- | --- | --- |
| AX-110013515 | TraesCS3B02G018800 | 1.477561029 | -0.041840733 | 1.226862805 | 1.393075385 |
|  | TraesCS3B02G019300 | 1.941553296 | 1.50047647 | 1.879413246 | 1.078312683 |
|  | TraesCS3B02G019500 | 2.448259612 | 2.107485822 | 2.396751703 | 2.181417481 |
|  | TraesCS3B02G019900 | 2.506891276 | 2.364420272 | 2.307621771 | 2.655637237 |
|  | TraesCS3B02G020200 | 0.278810724 | -0.719295144 | 0.478203092 | -2.303483514 |
|  | TraesCS3B02G020800 | 0.839610653 | 0.662629512 | 1.054426092 | -0.899785212 |
|  | TraesCS3B02G021400 | 1.988343307 | 1.808883502 | 1.876407282 | 1.836752446 |
|  | TraesCS3B02G021600 | 1.953187202 | 1.537547554 | 1.779053356 | 1.211342148 |
|  | TraesCS3B02G021700 | 1.375883284 | -1.424876732 | 1.667389072 | 0.46624291 |
|  | TraesCS3B02G022000 | 0.641733246 | -2.68322575 | -0.195186211 | -0.420673621 |
|  | TraesCS3B02G022300 | 1.852896155 | 0.961703907 | 1.490015221 | 1.432703518 |
|  | TraesCS3B02G022900 | 2.443620383 | 2.428154686 | 1.91370828 | 1.810766262 |
|  | TraesCS3B02G023200 | 0.758753724 | 0.152074144 | 1.047481304 | -0.153161368 |
|  | TraesCS3B02G023700 | 1.949837604 | 1.16628824 | 1.22908076 | -0.987960151 |
|  | TraesCS3B02G024900 | 1.483540591 | 1.235132779 | 1.430525119 | 0.625589741 |
|  | TraesCS3B02G025200 | 2.524780178 | 1.863195248 | 2.456541147 | 1.702727672 |
|  | TraesCS3B02G030900 | 0 | 0 | 0 | -0.393577667 |
|  | TraesCS3B02G031000 | 0 | 0 | 0 | -1.696275874 |
|  | TraesCS3B02G031300 | 0 | 0 | 0 | -0.116182762 |
|  | TraesCS3B02G032000 | 0.21623545 | 0 | -0.242025285 | 0.649086847 |
|  | TraesCS3B02G034100 | 1.709914512 | -0.381422251 | 1.684314223 | -1.336306237 |
|  | TraesCS3B02G034400 | 2.184136604 | 1.745664733 | 1.947642355 | 1.07809661 |
|  | TraesCS3B02G034500 | 2.055203188 | 1.642199509 | 1.928416394 | 1.626677762 |
|  | TraesCS3B02G035600 | 0.857331344 | 1.720302147 | 1.1646134 | -0.714359305 |
|  | TraesCS3B02G036200 | 1.40642732 | 0 | 0 | -0.457817195 |
|  | TraesCS3B02G036400 | 1.71224007 | -0.28872348 | 0 | 2.012557244 |
|  | TraesCS3B02G036900 | 2.663969758 | 2.323790771 | 1.964443485 | -0.959140161 |
|  | TraesCS3B02G037000 | 2.450780306 | 1.985878932 | 2.041981029 | 0.854542699 |
|  | TraesCS3B02G037200 | -0.02722527 | -0.754961963 | 0 | -4.874172079 |
|  | TraesCS3B02G037400 | 2.72137201 | 1.544667092 | -0.15460008 | 0.905106532 |
|  | TraesCS3B02G038300 | 2.450060054 | 0 | 0 | -4.518934481 |
|  | TraesCS3B02G038600 | 2.689707162 | 0.658837624 | 0 | 1.663561315 |
|  | TraesCS3B02G038700 | 2.620549394 | 1.836258292 | 1.320362164 | 1.067128875 |
|  | TraesCS3B02G039000 | -0.122532333 | 0 | 0 | 1.520425675 |
| AX-110922471 | TraesCS3B02G237100 | 1.752239122 | 1.895076746 | 1.916685886 | 0.927498097 |
|  | TraesCS3B02G237200 | 0.943860328 | 2.393782872 | 2.060108709 | 0.495610241 |
|  | TraesCS3B02G237400 | 1.953402408 | 1.774021842 | 1.83051031 | 1.819058404 |
|  | TraesCS3B02G237500 | 2.009926475 | 1.809155941 | 2.041504654 | 1.981296734 |
|  | TraesCS3B02G237600 | 1.235859024 | 0.603313784 | 1.183317935 | 0.841541836 |
|  | TraesCS3B02G237800 | 0 | 0 | -0.156883647 | -2.784994506 |
|  | TraesCS3B02G237900 | 2.187226858 | 1.67026602 | 1.922933501 | 1.64338139 |
|  | TraesCS3B02G238300 | 2.300754548 | 1.627737114 | 1.661711266 | 0.058713783 |
|  | TraesCS3B02G238600 | 2.170257317 | 1.866038821 | 1.995505431 | 1.339029297 |
|  | TraesCS3B02G238800 | 2.167990392 | 2.210685072 | 2.275686843 | 2.106992086 |
|  | TraesCS3B02G239000 | 1.93549354 | 1.553604692 | 1.789577857 | 1.380145471 |
|  | TraesCS3B02G239300 | 1.161507619 | 1.216023827 | 1.363218648 | 1.088255103 |
|  | TraesCS3B02G239400 | 2.232527522 | 1.958084138 | 1.963414611 | 0.855311024 |
|  | TraesCS3B02G239500 | 1.210481896 | 0.518327321 | 1.292516707 | 0.913051458 |
|  | TraesCS3B02G239700 | 1.913902193 | 1.800079327 | 1.804679667 | 1.057330369 |
|  | TraesCS3B02G239900 | 1.44590807 | 0.512136815 | 1.33747215 | 1.053712732 |
|  | TraesCS3B02G240000 | 1.657101221 | 1.624367024 | 1.750920871 | 1.364730667 |
|  | TraesCS3B02G240100 | 1.892892309 | 2.131973053 | 2.078408023 | 1.594999765 |
|  | TraesCS3B02G240300 | 2.151928253 | 1.49833522 | 1.496956584 | 2.036181184 |
|  | TraesCS3B02G240500 | 0 | 2.520803003 | 2.163483494 | 0.291907345 |
|  | TraesCS3B02G240800 | 1.720869533 | 1.398127637 | 1.658357492 | 1.137362814 |
|  | TraesCS3B02G240900 | 1.930755374 | 1.714702493 | 1.930103792 | 1.351194318 |
|  | TraesCS3B02G241000 | 2.551914748 | 2.44259275 | 2.475595148 | 2.744553371 |
|  | TraesCS3B02G241200 | 2.397158963 | 2.109750518 | 2.206391765 | 1.386039724 |
|  | TraesCS3B02G241300 | 0.887945832 | 0.498929445 | 0.878861303 | -0.161359244 |
|  | TraesCS3B02G241400 | 1.286832095 | 1.231372055 | 1.550619021 | 1.301475293 |
|  | TraesCS3B02G241500 | 0.860869244 | -0.287467485 | 0.392995018 | 0.208390703 |
|  | TraesCS3B02G241700 | 1.941714235 | 1.728874319 | 1.758904755 | 1.612068121 |
| AX-94729264 | TraesCS3D02G074400 | 2.694347411 | 3.063140734 | 2.899369124 | 2.915489373 |
|  | TraesCS3D02G074700 | 2.200734481 | 2.146651601 | 2.221689606 | 1.698122514 |
|  | TraesCS3D02G074800 | 1.353047296 | 0.310229589 | 0.538281552 | -2.801759597 |
|  | TraesCS3D02G074900 | 1.930198165 | 1.201088233 | 1.64184171 | 0.984789 |
|  | TraesCS3D02G075300 | 0 | 0 | 0 | -0.390496746 |
|  | TraesCS3D02G075700 | 1.426011999 | 1.15206595 | 1.35596423 | 1.275889236 |
|  | TraesCS3D02G075800 | 1.627389801 | 2.722251672 | 2.331715845 | 1.099198256 |
|  | TraesCS3D02G075900 | 2.934008322 | 2.47354546 | 2.946683031 | 2.345386847 |
|  | TraesCS3D02G076500 | 1.452576654 | 1.34470251 | 1.354146293 | 0.770204972 |
|  | TraesCS3D02G077100 | 0.844341121 | 0.702462299 | 0.865520903 | 0.741616332 |
|  | TraesCS3D02G077200 | 2.276632366 | 2.130064419 | 2.047882496 | 1.699796529 |
|  | TraesCS3D02G077300 | 0 | 0 | 0 | 2.25965528 |
|  | TraesCS3D02G077500 | 0 | 1.758355922 | 1.647794473 | 1.964342718 |
|  | TraesCS3D02G078500 | 0 | 0 | 0 | 2.016795458 |
|  | TraesCS3D02G078700 | 2.623479317 | 2.301024169 | 2.332639181 | 1.618176966 |
|  | TraesCS3D02G079000 | 1.503873652 | 2.023364054 | 1.89076387 | 1.416652939 |
|  | TraesCS3D02G079200 | 1.44574065 | 1.485557918 | 1.6271962 | 1.293135826 |
|  | TraesCS3D02G079600 | 1.517177548 | 1.381889123 | 1.576227895 | 1.939528948 |
|  | TraesCS3D02G079800 | 2.490431518 | 2.169174014 | 2.222688721 | 1.839918909 |
|  | TraesCS3D02G080300 | 0.887481175 | 0.419389949 | 0.699727741 | 0.373714559 |
|  | TraesCS3D02G080900 | 0 | 0 | -0.708375015 | 0.790909381 |
|  | TraesCS3D02G081400 | 1.376423991 | -0.005004209 | 1.298539873 | 0.436901016 |
|  | TraesCS3D02G082200 | -0.553398594 | -0.650630652 | 0.083145079 | -0.780086659 |
|  | TraesCS3D02G082300 | 2.571461475 | 2.331755034 | 2.468798217 | 2.480830926 |
|  | TraesCS3D02G082400 | 1.148445463 | 1.173886638 | 1.227015383 | 0.022450339 |
|  | TraesCS3D02G082900 | -0.267412731 | 1.452038749 | 1.290070545 | -1.136585501 |
|  | TraesCS3D02G083200 | 1.62787603 | 0.514226262 | 1.152457867 | 0.620857668 |
|  | TraesCS3D02G083400 | -1.22382523 | 2.443549054 | 1.927890853 | -1.148701392 |
|  | TraesCS3D02G083700 | 1.883810641 | 2.678723965 | 2.274516392 | 0.719228414 |
|  | TraesCS3D02G083800 | 1.52363298 | 1.415714769 | 1.552683398 | 1.289151191 |
|  | TraesCS3D02G084600 | 0 | 0 | -0.590655786 | 1.508563047 |
|  | TraesCS3D02G084900 | 0 | 1.754905328 | 0.276124264 | 1.159918727 |
|  | TraesCS3D02G085400 | 2.095784833 | 1.901730203 | 1.98545925 | 1.682091436 |
|  | TraesCS3D02G085500 | 1.843144575 | 1.666975081 | 1.792869437 | 1.640709751 |
|  | TraesCS3D02G085600 | 3.024992203 | 2.713825267 | 2.85405577 | 2.781179329 |
|  | TraesCS3D02G085700 | 0.59563697 | 1.546504831 | 1.432676688 | -0.340745516 |
|  | TraesCS3D02G085900 | 0 | 0 | 0.756031127 | -0.262614817 |
|  | TraesCS3D02G086700 | -0.119138788 | 0.063455394 | 0.079056467 | -1.254903835 |
|  | TraesCS3D02G086800 | 0 | 0 | 0.894011393 | 0.411656944 |
|  | TraesCS3D02G086900 | 2.124194705 | 2.149892144 | 2.2375598 | 1.976021105 |
|  | TraesCS3D02G087000 | 1.088979326 | -0.025789719 | 1.008906245 | -0.123260231 |
|  | TraesCS3D02G087100 | 0.974174415 | 0.738975611 | 1.055711402 | 0.338539861 |
|  | TraesCS3D02G087200 | 0.812022789 | 0.443412942 | 0.844180829 | 0.504648182 |
|  | TraesCS3D02G087300 | 2.375453993 | 2.742480302 | 2.373338067 | 1.713574117 |
|  | TraesCS3D02G087400 | 1.568470661 | 1.962673666 | 2.040921641 | 0.599431859 |
|  | TraesCS3D02G087500 | 1.970232958 | 1.423442728 | 1.081106694 | 1.464456047 |
|  | TraesCS3D02G087900 | 1.612825558 | 1.641469599 | 1.508364875 | 0.783167311 |
|  | TraesCS3D02G088900 | 2.349013761 | 1.930077971 | 2.554388345 | 1.812308097 |
|  | TraesCS3D02G089000 | 1.35856108 | 1.397606165 | 1.437277346 | 1.225516727 |
|  | TraesCS3D02G089200 | 0.57494176 | 1.363983185 | 0.936230105 | -0.36935021 |
| AX-108912427 | TraesCS4A02G425000 | 1.185440146 | 0.69282279 | 1.416796436 | 1.369065597 |
|  | TraesCS4A02G425100 | 0.990767363 | 1.154868983 | 1.374398151 | 1.409658251 |
|  | TraesCS4A02G425300 | 1.982023749 | 0.812626906 | 1.275159959 | 0.823373043 |
|  | TraesCS4A02G426100 | 1.768420786 | 2.346936381 | 1.91508805 | 0.9327403 |
|  | TraesCS4A02G427500 | 2.133101564 | 1.665661572 | 1.95026658 | 1.737629814 |
|  | TraesCS4A02G428500 | 1.75063499 | 0.78853093 | 1.372838521 | 1.038679569 |
|  | TraesCS4A02G428600 | 1.360861474 | 0.700018565 | 0.95040517 | 1.220306728 |
|  | TraesCS4A02G428700 | 0.570105227 | -0.032195051 | 0.002153418 | -0.751629155 |
|  | TraesCS4A02G428900 | 2.674425293 | 2.609236931 | 2.645295698 | 2.771954566 |
|  | TraesCS4A02G429200 | 0.98091072 | 0.611839195 | 1.082829091 | -0.019672818 |
|  | TraesCS4A02G429300 | 1.179043998 | 0.324572857 | 0.803846937 | 0.589847302 |
|  | TraesCS4A02G429400 | 2.470275274 | 2.101293361 | 2.332088597 | 1.892406847 |
|  | TraesCS4A02G430000 | 0 | 0 | 0 | 1.921681232 |
|  | TraesCS4A02G430300 | 0.633108348 | -0.478468317 | 0.935031081 | -1.591585796 |
|  | TraesCS4A02G430500 | 0 | 0 | 0 | -2.628928097 |
|  | TraesCS4A02G431200 | 0 | 0 | 0 | 2.368412862 |
|  | TraesCS4A02G431800 | 0 | 0 | 0 | 1.102221662 |
|  | TraesCS4A02G431900 | 0 | 0 | 0 | 1.191053777 |
|  | TraesCS4A02G432000 | 0 | 0 | 0 | 0.397276245 |
|  | TraesCS4A02G432200 | 0.014743314 | -0.410657046 | -0.499856974 | -1.749081334 |
|  | TraesCS4A02G432300 | 1.884811341 | 0.99686946 | 0.152009732 | -1.042185032 |
|  | TraesCS4A02G432400 | 1.901554628 | 1.614123154 | 1.800693282 | 1.629958324 |

Note: The expression profiles of the high-confidence candidate genes were obtained from the public database of Wheat Expression Browser (http://www.wheat-expression.com) and were performed log_2_ conversion.

**Table S6.** Number of superior and inferior alleles across 11 significantly associated SNPs identified by three statistical models in the genome of 207 wheat varieties.

| **Accession ID.** | **No. of superior alleles** | **No. of inferior alleles** | **Ca_BLUP** |
| --- | --- | --- | --- |
| L001 | 2 | 6 | 326.91 |
| L002 | 1 | 7 | 333.15 |
| L003 | 1 | 7 | 336.07 |
| L004 | 0 | 8 | 328.61 |
| L005 | 1 | 7 | 331.79 |
| L006 | 1 | 8 | 330.22 |
| L007 | 2 | 6 | 336.27 |
| L008 | 2 | 6 | 332.95 |
| L009 | 2 | 5 | 334.06 |
| L010 | 0 | 8 | 331.30 |
| L011 | 0 | 8 | 332.93 |
| L012 | 2 | 6 | 332.91 |
| L013 | 3 | 5 | 324.37 |
| L014 | 1 | 8 | 326.51 |
| L015 | 1 | 7 | 325.46 |
| L016 | 2 | 7 | 325.17 |
| L017 | 5 | 3 | 329.03 |
| L018 | 4 | 5 | 326.43 |
| L019 | 0 | 7 | 327.08 |
| L020 | 0 | 7 | 325.71 |
| L021 | 1 | 7 | 325.37 |
| L022 | 0 | 8 | 328.44 |
| L023 | 1 | 7 | 327.12 |
| L024 | 3 | 6 | 325.35 |
| L025 | 2 | 6 | 322.42 |
| L026 | 2 | 7 | 327.34 |
| L027 | 1 | 8 | 326.64 |
| L028 | 1 | 7 | 328.64 |
| L029 | 1 | 8 | 323.75 |
| L030 | 0 | 8 | 327.69 |
| L031 | 1 | 7 | 322.16 |
| L032 | 1 | 7 | 322.72 |
| L033 | 2 | 7 | 323.09 |
| L034 | 1 | 7 | 322.81 |
| L035 | 1 | 7 | 326.86 |
| L036 | 1 | 7 | 326.49 |
| L037 | 5 | 4 | 325.99 |
| L038 | 1 | 8 | 325.62 |
| L039 | 1 | 8 | 325.00 |
| L040 | 2 | 7 | 325.05 |
| L041 | 2 | 7 | 327.11 |
| L042 | 3 | 5 | 328.43 |
| L043 | 1 | 8 | 325.83 |
| L044 | 2 | 6 | 326.77 |
| L045 | 1 | 7 | 328.69 |
| L046 | 0 | 9 | 326.78 |
| L047 | 2 | 6 | 328.11 |
| L048 | 2 | 7 | 332.77 |
| L049 | 1 | 7 | 328.91 |
| L050 | 2 | 6 | 331.15 |
| L051 | 1 | 8 | 330.99 |
| L052 | 2 | 6 | 329.70 |
| L053 | 1 | 7 | 327.19 |
| L054 | 2 | 7 | 327.34 |
| L055 | 1 | 7 | 329.66 |
| L056 | 3 | 5 | 328.20 |
| L057 | 2 | 6 | 328.73 |
| L058 | 1 | 7 | 327.50 |
| L059 | 4 | 5 | 328.10 |
| L060 | 0 | 8 | 327.77 |
| L061 | 3 | 6 | 327.78 |
| L062 | 1 | 7 | 326.53 |
| L063 | 3 | 5 | 325.09 |
| L064 | 1 | 7 | 328.86 |
| L065 | 2 | 6 | 326.46 |
| L066 | 1 | 7 | 327.73 |
| L067 | 1 | 7 | 325.89 |
| L068 | 1 | 7 | 328.06 |
| L069 | 2 | 7 | 327.41 |
| L070 | 2 | 7 | 329.07 |
| L071 | 1 | 7 | 326.90 |
| L072 | 3 | 6 | 325.36 |
| L073 | 2 | 7 | 330.75 |
| L074 | 3 | 5 | 329.38 |
| L075 | 1 | 7 | 327.38 |
| L076 | 1 | 7 | 331.62 |
| L077 | 1 | 7 | 327.30 |
| L078 | 1 | 7 | 328.57 |
| L079 | 2 | 6 | 330.42 |
| L080 | 1 | 7 | 332.56 |
| L081 | 2 | 6 | 333.36 |
| L082 | 1 | 8 | 332.73 |
| L083 | 1 | 8 | 329.51 |
| L084 | 1 | 8 | 330.55 |
| L085 | 3 | 4 | 331.02 |
| L086 | 1 | 7 | 330.38 |
| L087 | 2 | 7 | 331.58 |
| L088 | 4 | 5 | 332.38 |
| L089 | 1 | 8 | 337.46 |
| L090 | 2 | 6 | 338.03 |
| L091 | 4 | 4 | 332.73 |
| L092 | 1 | 8 | 329.98 |
| L093 | 0 | 8 | 327.06 |
| L094 | 3 | 5 | 333.04 |
| L095 | 1 | 7 | 333.96 |
| L096 | 6 | 3 | 333.92 |
| L097 | 3 | 5 | 330.59 |
| L098 | 1 | 7 | 327.07 |
| L099 | 4 | 5 | 334.80 |
| L100 | 7 | 1 | 346.02 |
| L101 | 6 | 3 | 344.83 |
| L102 | 4 | 5 | 336.52 |
| L103 | 4 | 5 | 341.75 |
| L104 | 7 | 2 | 340.18 |
| L105 | 6 | 3 | 342.37 |
| L106 | 6 | 3 | 341.80 |
| L107 | 5 | 4 | 335.52 |
| L108 | 6 | 3 | 334.53 |
| L109 | 6 | 3 | 335.51 |
| L110 | 6 | 3 | 331.78 |
| L111 | 7 | 2 | 339.08 |
| L112 | 3 | 6 | 330.03 |
| L113 | 4 | 5 | 335.76 |
| L114 | 5 | 4 | 331.14 |
| L115 | 8 | 1 | 335.28 |
| L116 | 8 | 1 | 334.26 |
| L117 | 6 | 3 | 331.95 |
| L118 | 6 | 3 | 339.85 |
| L119 | 5 | 4 | 334.95 |
| L120 | 5 | 4 | 333.29 |
| L121 | 5 | 4 | 337.37 |
| L122 | 6 | 3 | 332.05 |
| L123 | 6 | 3 | 341.72 |
| L124 | 5 | 4 | 338.54 |
| L125 | 7 | 2 | 340.05 |
| L126 | 5 | 4 | 343.79 |
| L127 | 5 | 4 | 342.78 |
| L128 | 4 | 5 | 334.29 |
| L129 | 6 | 2 | 340.68 |
| L130 | 7 | 2 | 346.20 |
| L131 | 4 | 5 | 342.19 |
| L132 | 5 | 4 | 334.49 |
| L133 | 5 | 4 | 336.79 |
| L134 | 7 | 2 | 348.08 |
| L135 | 6 | 3 | 342.87 |
| L136 | 6 | 3 | 346.85 |
| L137 | 5 | 4 | 342.63 |
| L138 | 7 | 2 | 344.22 |
| L139 | 5 | 4 | 331.83 |
| L140 | 4 | 5 | 334.31 |
| L141 | 3 | 6 | 330.98 |
| L142 | 4 | 5 | 325.44 |
| L143 | 5 | 4 | 329.96 |
| L144 | 5 | 4 | 333.26 |
| L145 | 4 | 5 | 331.32 |
| L146 | 4 | 5 | 333.44 |
| L147 | 4 | 5 | 331.87 |
| L148 | 4 | 5 | 332.65 |
| L149 | 2 | 6 | 326.16 |
| L150 | 4 | 5 | 328.75 |
| L151 | 5 | 4 | 326.75 |
| L152 | 2 | 6 | 328.31 |
| L153 | 5 | 4 | 328.52 |
| L154 | 5 | 4 | 328.55 |
| L155 | 4 | 4 | 332.73 |
| L156 | 3 | 4 | 324.81 |
| L157 | 4 | 5 | 329.43 |
| L158 | 6 | 3 | 325.43 |
| L159 | 7 | 2 | 327.62 |
| L160 | 4 | 5 | 335.25 |
| L161 | 5 | 3 | 329.76 |
| L162 | 3 | 6 | 329.67 |
| L163 | 4 | 4 | 323.40 |
| L164 | 4 | 5 | 328.15 |
| L165 | 4 | 5 | 327.80 |
| L166 | 4 | 5 | 326.02 |
| L167 | 3 | 5 | 339.56 |
| L168 | 2 | 7 | 324.36 |
| L169 | 1 | 7 | 326.42 |
| L170 | 3 | 6 | 328.58 |
| L171 | 5 | 4 | 331.27 |
| L172 | 4 | 5 | 325.25 |
| L173 | 4 | 5 | 324.81 |
| L174 | 5 | 4 | 333.59 |
| L175 | 3 | 4 | 327.29 |
| L176 | 4 | 5 | 326.52 |
| L177 | 2 | 7 | 326.90 |
| L178 | 4 | 5 | 327.75 |
| L179 | 6 | 3 | 324.73 |
| L180 | 3 | 6 | 320.96 |
| L181 | 3 | 6 | 326.13 |
| L182 | 2 | 7 | 328.76 |
| L183 | 5 | 4 | 325.29 |
| L184 | 4 | 5 | 324.75 |
| L185 | 3 | 6 | 327.60 |
| L186 | 6 | 3 | 326.61 |
| L187 | 4 | 5 | 325.16 |
| L188 | 4 | 5 | 328.59 |
| L189 | 5 | 4 | 329.45 |
| L190 | 4 | 5 | 324.96 |
| L191 | 5 | 4 | 329.38 |
| L192 | 6 | 3 | 330.96 |
| L193 | 6 | 3 | 333.95 |
| L194 | 6 | 3 | 329.99 |
| L195 | 6 | 3 | 336.04 |
| L196 | 7 | 2 | 334.21 |
| L197 | 4 | 5 | 334.48 |
| L198 | 5 | 3 | 334.62 |
| L199 | 5 | 4 | 333.10 |
| L200 | 4 | 5 | 330.38 |
| L201 | 4 | 5 | 326.91 |
| L202 | 5 | 4 | 332.36 |
| L203 | 4 | 5 | 334.18 |
| L204 | 6 | 3 | 328.12 |
| L205 | 3 | 6 | 332.70 |
| L206 | 5 | 4 | 330.57 |
| L207 | 3 | 6 | 327.82 |
